# Allosteric Partial Inhibition of Monomeric Proteases. Sulfated Coumarins Induce Regulation, not just Inhibition, of Thrombin

**DOI:** 10.1038/srep24043

**Published:** 2016-04-07

**Authors:** Stephen Verespy III, Akul Y. Mehta, Daniel Afosah, Rami A. Al-Horani, Umesh R. Desai

**Affiliations:** 1 Institute for Structural Biology, Drug Discovery and Development, Virginia Commonwealth University, Richmond, Virginia, USA; 2 Department of Chemistry, Virginia Commonwealth University, Richmond, Virginia, USA; 3 Department of Medicinal Chemistry, Virginia Commonwealth University, Richmond, Virginia, USA

## Abstract

Allosteric partial inhibition of soluble, monomeric proteases can offer major regulatory advantages, but remains a concept on paper to date; although it has been routinely documented for receptors and oligomeric proteins. Thrombin, a key protease of the coagulation cascade, displays significant conformational plasticity, which presents an attractive opportunity to discover small molecule probes that induce sub-maximal allosteric inhibition. We synthesized a focused library of some 36 sulfated coumarins to discover two agents that display sub-maximal efficacy (~50%), high potency (<500 nM) and high selectivity for thrombin (>150-fold). Michaelis-Menten, competitive inhibition, and site-directed mutagenesis studies identified exosite 2 as the site of binding for the most potent sulfated coumarin. Stern-Volmer quenching of active site-labeled fluorophore suggested that the allosteric regulators induce intermediate structural changes in the active site as compared to those that display ~80–100% efficacy. Antithrombin inactivation of thrombin was impaired in the presence of the sulfated coumarins suggesting that allosteric partial inhibition arises from catalytic dysfunction of the active site. Overall, sulfated coumarins represent first-in-class, sub-maximal inhibitors of thrombin. The probes establish the concept of allosteric partial inhibition of soluble, monomeric proteins. This concept may lead to a new class of anticoagulants that are completely devoid of bleeding.

Allosterism induced by small or large molecule effectors is increasingly being perceived as a new paradigm to understand molecular pathways and discover new therapeutics^1^,^2^. It refers to modulating a protein’s biological function through allosteric (distal) sites, rather than it’s orthosteric (active) site. Small molecules that target allosteric sites have become extremely useful probes for advancing chemical biology and drug discovery projects^1^,^2^,^3^. A range of allosteric targets are described in the literature including receptors or membrane-bound proteins^3^,^4^,^5^,^6^, kinases^7^,^8^, and proteases^1^,^9^,^10^,^11^,^12^.

Intrinsically, allostery offers some major advantages over orthostery. Whereas orthosteric sites between related proteins/enzymes are similar, e.g., trypsin-like serine proteases^10^,^13^, allosteric sites are typically less conserved^1^,^5^,^14^. Allostery can afford dramatic changes in the type of biological function, e.g., procoagulation to anticoagulation, while orthostery can afford only a reduction in biological activity, e.g., inhibition of catalytic activity. Finally, allostery presents two parameters – potency (*IC*_50_) and efficacy (% ΔY) – for regulation of activity, whereas orthostery presents only one, i.e., potency, for regulation (Fig. 1)^15^. More specifically, allosteric induction of function depends on the cooperativity between the distal and active sites, which is expected to be sensitive to the structure of the allosteric ligand, and thereby, may exhibit variable levels of cooperativity. Alternatively, whereas one allosteric ligand may exhibit maximum possible conformational change in the active site at saturation (ΔY = 80–100%), another may exhibit only partial conformational change at saturation (ΔY = 30–70%). Such a phenomenon is not possible for orthosteric ligands and thus, allostery offers better regulation possibilities. Yet, partial or sub-maximal allosteric regulators, more often described as partial agonists or antagonists of receptors^16^, have been extensively described for receptors/membrane-bound proteins^16^,^17^ and oligomeric, secreted proteins^18^, but remain unknown for soluble, monomeric proteins, e.g., proteases.

A key protease of special interest to the field of blood coagulation is thrombin. Thrombin is a trypsin-like monomeric protease that functions as a procoagulant by cleaving soluble fibrinogen into fibrin and as an anticoagulant by activating protein C in the presence of thrombomodulin^19^,^20^. In addition, thrombin recognizes multiple natural substrates, e.g., factors V, VIII, XI and others, and unnatural agents, e.g., hirudin. Thrombin exhibits considerable structural plasticity by sampling a number of conformational forms in its ground state^19^,^20^,^21^, which are in equilibrium with each other (Fig. 1). Theoretically, this equilibrium can be perturbed by appropriate small molecules/ions. In fact, Na^+^ ion is recognized as a natural modulator of thrombin activity. Na^+^ binding to thrombin enhances substrate hydrolysis, whereas absence of Na^+^ shifts the equilibrium to a “slow” form^22^.

We reasoned that thrombin is an ideal monomeric protease for discovering small molecule probes that display sub-maximal conformational change at saturation. Such probes would induce only a 50% cleavage of substrates at saturation (ΔY = ~30–70%) and afford the possibility of better regulation of the protease. For example, current orthosteric inhibitors of thrombin (dabigatran, argatroban, etc.) inhibit thrombin with maximal efficacy (ΔY = 100%), which contributes to enhanced risk of bleeding^23^,^24^. In contrast, we predict that sub-maximal inhibitors of thrombin will retain some hemostatic potential, which will prevent bleeding consequences^25^. However, such small molecule sub-maximal allosteric regulators of thrombin remain unknown.

We have earlier studied a number of sulfated non-saccharide glycosaminoglycan allosteric modulators (NSGMs) of thrombin including sulfated benzofurans^26^,^27^,^28^,^29^ and sulfated low molecular weight lignins^30^,^31^. These NSGMs display inhibition efficacy in the range of 70–100% and are more accurately “inhibitors” rather than “regulators”. In this work, we present sulfated coumarins as novel allosteric regulators of thrombin with excellent potency and high selectivity for thrombin. Kinetic and thermodynamic studies suggest that inhibition arises from a sub-maximal change in the conformation/structure of the active site. Thus, sulfated coumarins represent first-in-class, allosteric regulators of thrombin and establish the idea that it is possible to realize sub-maximal allosteric regulators of soluble, monomeric proteins.

## Results & Discussion

### Synthesis of a Library of Sulfated Coumarins

In our quest to discover allosteric partial regulators, especially of thrombin, we decided to study the sulfated coumarin scaffold. Our earlier work on designing/discovering allosteric modulators of thrombin led to the sulfated quinazolinone and benzofuran scaffolds, which exhibited allosterism, but induced full inhibition (ΔY = 80–100%) at saturation^26^,^27^,^28^,^29^. Although sulfated coumarins are similar to sulfated quinazolinones and sulfated benzofurans studied earlier in carrying one or more sulfate groups, the aromatic scaffold is fundamentally different. The unique electrotopological nature^31^ of coumarins coupled with their synthetic accessibility^32^ led us to investigate whether this scaffold can provide allosteric partial regulator(s) of thrombin.

Sulfated coumarins were synthesized using either a protection–deprotection strategy or a selective reaction strategy, which enabled the generation of the desired number of free phenolic/alcoholic groups that can be sulfated in the final step using sulfur trioxide–trialkylamine complex^27^,^28^,^29^,^33^. One advantage here was that quite a few naturally occurring coumarins were available, which could be directly sulfated under microwave-assisted conditions, as described previously^34^,^35^. This led to the synthesis of monomeric sulfated coumarins **1a**–**1d** and **2a–2v** (Fig. 2) in high yields (82–99%) and high purity (>95%, see Supplementary Fig. S1). Likewise, naturally occurring *O*-methylated coumarins were deprotected utilizing BBr_3_ and then sulfated to synthesize **2x–2z** in yields of 91–94% (Fig. 2 and Supplementary Fig. S2).

To synthesize dimeric analogs of sulfated coumarins, a copper-assisted azide-alkyne cycloaddition (CuAAC) was employed, in which selective S_N_2-based coupling at the most reactive phenol (7-OH group) led to synthesis of head-to-head dimers **3a–3f** in yields of 72–90% (Fig. 2 and Supplementary Fig. S3). The structures of these agents were confirmed through detailed 2D NMR experiments that unambiguously established the position of reaction under these conditions (see Supplementary Figs S4 and S5). Likewise, the head-to-tail dimer **3g** and **3h** was also synthesized utilizing the conditions developed for the head-to-head dimers with appropriate modifications in excellent yields (Fig. 2). The head-to-tail structure of **3g** was also confirmed through 2D NMR (Supplementary Fig. S6). Overall, each member of the sulfated coumarin library could be synthesized in less than 6 steps (see Supplementary Information).

This is the first synthesis of a NSGM library based on the coumarin scaffold. The 36 member library contained different locations of the 1 or 2 sulfate group(s), different types of substitutions (-Me, -Ph, -Cl, -Br, etc.), different size of molecules (monomer or dimer), different linker length (n = 3 or 4), and different geometry of constituent units (“head-to-head” or “head-to-tail”) (see Fig. 2). Thus, although the library is not large, it presents sufficient possibilities of discovering probes of partial allosterism.

### Inhibition and Regulation of Coagulation Factors

The library of sulfated coumarins was first screened against thrombin, as well as related coagulation factors, factor Xa and factor XIa, at a high concentration (250 μM) using appropriate chromogenic substrates, as described earlier^29^,^33^,^36^. Eleven of the 36 sulfated coumarins displayed reasonable inhibition of thrombin and were selected for further characterization. Figure 3A shows the dose–response profiles of selected sulfated coumarins and a non-sulfated precursor. Supplementary Table S1 lists the half-maximal inhibitory concentration (*IC*_50_), efficacy of inhibition (∆Y) and Hill slope (HS) for each molecule calculated through non-linear regression analysis (equation 1). In terms of structure–function relationships, the results showed a highly sensitive thrombin inhibition response to the structure of sulfated coumarins. Very few monomeric sulfated coumarins inhibited thrombin (only **2e**, **2k** and **2p**), in contrast to nearly all dimeric sulfated coumarins that displayed good inhibition potencies (*IC*_50_ < 20 μM). Only those monomeric NSGMs that contained an aromatic group at the 4-position were active, e.g., **2e**, **2k** and **2p**, but not all 4-aryl derivatives were active, e.g., **2d**, **2l**, **2o**, **2w**, **2x** and **2y** (see Supplementary Table S1). One or more halogen atoms were also present on the monomeric scaffold. Each of the active monomers contained one sulfate group, preferably at the 7-position, and removal of this sulfate or introduction of an additional sulfate group (e.g., **2y**, see Fig. 2) led to complete absence of inhibition potential.

The higher reactivity of the 7-OH gave 7–7-linked dimers **3a**–**3f**, which contained sulfation at the 4′-hydroxyphenyl position (see Fig. 2). Although structurally, the dimers cannot be considered as strict analogs of the monomers, the ease of their synthesis led us to study these variants. Each of the dimers studied inhibited thrombin with a higher potency than monomers (Fig. 3A and Supplementary Table S1). Of these, **3a** and **3b** were most potent (*IC*_50_ ≤ 1 μM). Interestingly, **3a** and **3b**, and not any other dimer, contain 4-phenyl substitution, which mimics the structure–activity relationship observed for monomers. To assess whether an alternative analog would yield similar activity, we synthesized **3g** (see Fig. 2). Dimer **3g** possesses only one sulfate, opposed to the other six dimers, which have two. Interestingly, **3g** inhibited thrombin with an *IC*_50_ of 180 nM against thrombin (Supplementary Table S1), the highest potency observed in the group. More importantly, it is selective for thrombin as evidenced by its inhibition potencies against closely related coagulation proteases factors Xa and XIa (*IC*_50_ > 30 μM, Supplementary Table S1).

Structurally, dimer **3g** shows a couple of similarities with active monomer **2k**. Both contain 4-aryl and *ortho-*/*para*- dichloro substitution. Considering that **3g** and **2k** are the most potent molecules in their respective groups, it is likely that these substitutions contribute to enhancing potency against thrombin. We speculate that hydrophobic forces play a significant role in targeting thrombin. This is further supported by the observation that **3g**, which contains only one sulfate group, is more potent than the rest of dimers, which contain two sulfate groups. To assess, whether removal of the lone sulfate at the 4′-hydroxyphenyl position in **3g** further enhances potency, we studied non-sulfated precursor **3h** (Fig. 2). Even high concentrations of **3h** did not inhibit thrombin, or any other protease tested (Fig. 3A). This implies that the lone sulfate of **3g** is critical for thrombin inhibition. Yet, this does not conclusively imply that sulfation at the 4′-hydroxylphenyl is the most optimal position. Many analogs of **3g** will need to be studied to establish the structure-activity relationships around this scaffold.

To characterize **3g**′s thermodynamic affinity for thrombin, we used fluorescein-labeled (*f* FPRCK-) human thrombin and followed its fluorescence as a function of **3g** levels, as also performed for other sulfated ligands of coagulation factors^37^,^38^. As shown in Fig. 3B, a characteristic loss in fluorescence of thrombin was observed, which could be fitted using the quadratic binding equation (equation 4) to obtain a *K*_D_ of 143 ± 14 nM and ΔF_MAX_ of ~28%. Thus, the thermodynamic affinity of **3g** is in the same range as its inhibition potency. Finally, inhibitor **3g** also displays excellent selectivity for thrombin in comparison to that for factor XIa (~150-fold) and factor Xa (~800-fold) (Fig. 3C) highlighting its putative value in studying thrombin allostery and function.

The high potency or selectivity of inhibition noted for **3g** was not the most interesting discovery from this study. It was the variable efficacy (ΔY) of thrombin inhibition. All of sulfated coumarins assayed displayed ΔYs ranging from ~20–70% (Fig. 3A and Supplementary Table S1), indicating that this is a generic property of the sulfated coumarin scaffold. The most potent sulfated coumarin **3g** displayed an efficacy of 47% and a Hill slope of 1.2. Other reasonably potent agents displayed ∆Y of 73% (**3a**), 58% (**3b**) and 60% (**3c**). In contrast, sulfated benzofurans and sulfated quinazolinones studied earlier display >80% inhibition^27^,^28^,^29^ or no inhibition^33^ of thrombin. Likewise, other sulfated molecules described in the literature that inhibit a number of other coagulation factors, e.g., factor Xa^30^,^39^, factor XIa^33^,^38^, or plasmin^40^,^41^, also do not display variable efficacies. Interestingly, the current sulfated coumarins inhibit factors Xa and XIa, which are structurally homologous to thrombin, with efficacies of 80–100% (Fig. 3B and Supplementary Table S1). This implies that sulfated coumarins, in principle, belong to a different class of thrombin inhibitors and present a unique capability of thrombin regulation.

### Mechanism of Thrombin Inhibition by Sulfated Coumarins

To identify the kinetic mechanism of thrombin inhibition by sulfated coumarins, especially **3g**, we performed Michaelis-Menten kinetic studies using Spectrozyme TH as the substrate. Figure 4A shows the substrate hydrolysis profiles in the presence of **3g**. As the concentration of **3g** increased, the *V*_MAX_ decreased from 57.5 to 31.0 mAU/min, whereas the *K*_M_ remained relatively constant (Supplementary Table S2). These results suggest that **3g**, and most probably other sulfated coumarins, non-competitively inhibit thrombin. Further, even at saturating levels of **3g**, the *V*_MAX_ did not reduce beyond ~50%, further confirming the partial inhibition characteristic of these inhibitors.

### Site of 3g Binding to Thrombin

**Figure 1:**
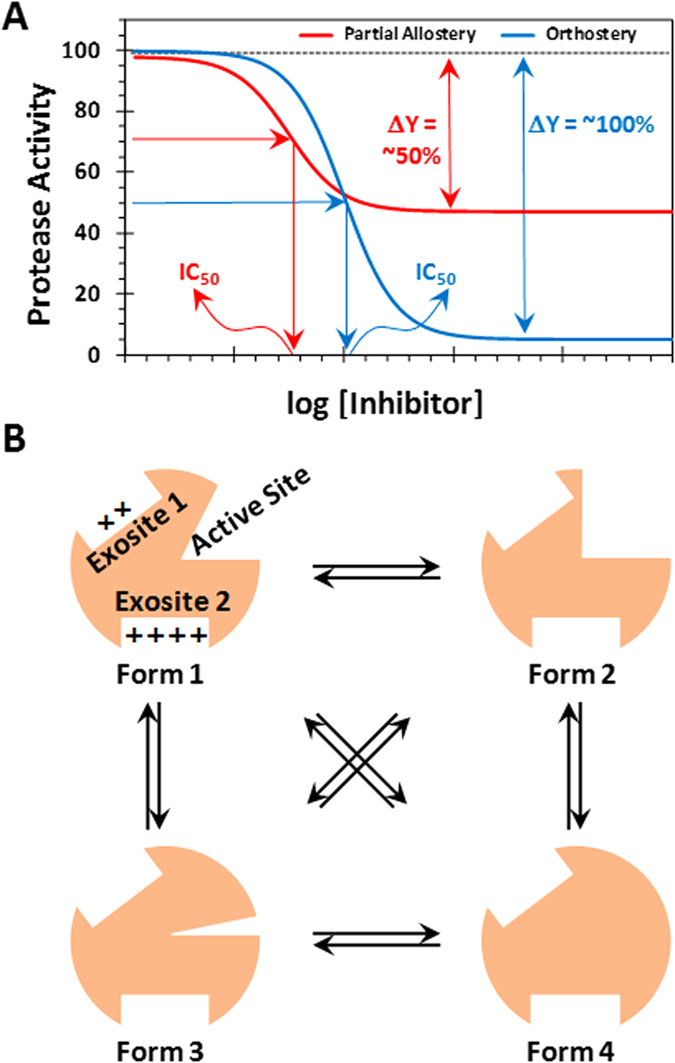
Allosterism and inhibition. (**A**) Allostery offers several major advantages over orthostery. Whereas both allostery and orthostery offer potency (*IC*_50_) for regulating inhibition, allostery (especially partial allostery) offers efficacy (% ΔY) in addition for controlling protease inhibition. (**B**) Thrombin, a soluble monomeric protease, is a highly plastic enzyme that displays multiple conformational isoforms in its ground state^19^,^21^,^57^. These conformational forms, labeled 1 through 4, are in equilibrium, which can theoretically be enriched through small molecules that bind at an allosteric site. Such allosteric agents may display partial inhibition of the protease at saturation, a phenomenon not known for monomeric proteases.

**Figure 2:**
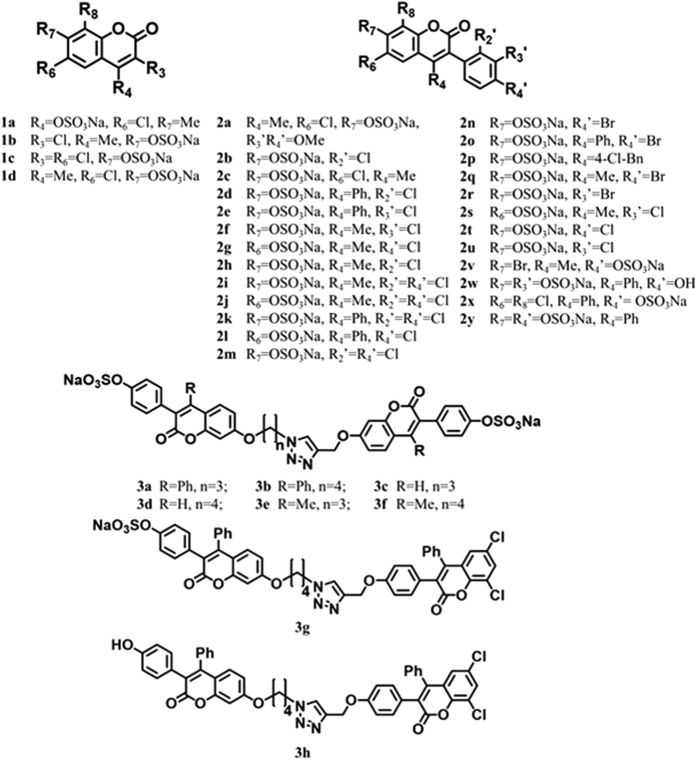
Structures of sulfated coumarins synthesized in this study to discover partial allosteric inhibitors of thrombin. **1a–d** are monomeric agents composed of only the coumarin core; **2a–y** are monomeric sulfated coumarins with 3-phenyl substitution; **3a**–**f** are dimeric “head-to-head” agents; and **3g** is the dimeric “head-to-tail” sulfated coumarin. Note: When a particular substituent **R**_***i***_ = H, it is not listed in above descriptions.

**Figure 3:**
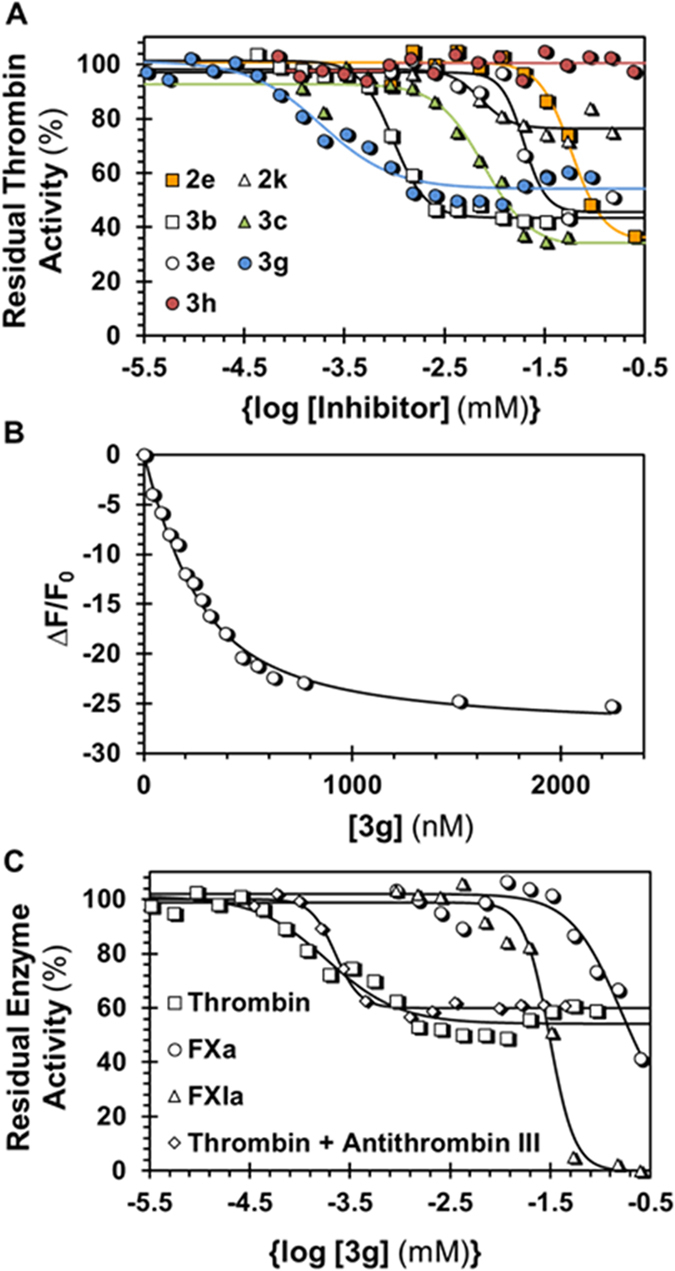
Sulfated coumarins as inhibitors of thrombin. (**A**) Dose-response profiles of selected sulfated coumarins (and non-sulfated **3g**) against thrombin in 20 mM Tris-HCl, 100 mM NaCl, 2.5 mM CaCl_2_, 0.1% PEG 8000, pH 7.4 at 25 °C. (**B**) Spectrofluorometric measurement of the affinity of **3g** for thrombin labeled at the active site using fluorescein-FPRCK (λ_EX_ = 490 nm, λ_EM_ = 525 nm). Solid lines represent nonlinear regression fitting using quadratic equation 4 to calculate the *K*_D_ of binding. (**C**) Dose-response profiles of **3g** against factor Xa and factor XIa with comparison against thrombin. Also shown is the profile for **3g** inhibition of thrombin in the presence of 100 nM antithrombin. Solid lines represent sigmoidal dose-response fits using equation 1 to calculate *IC*_50_, ∆Y and HS.

**Figure 4:**
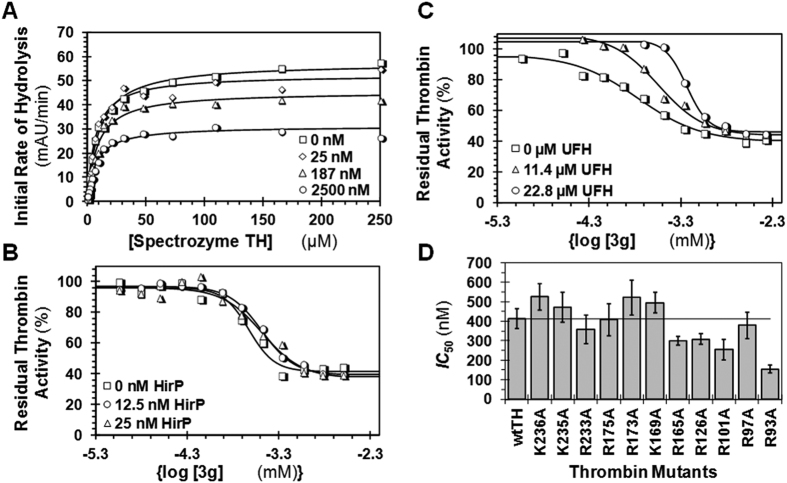
Site of 3g binding onto thrombin. (**A**) Michaelis-Menten kinetics Spectrozyme TH hydrolysis by thrombin in the presence of **3g**. The initial rate of hydrolysis at substrate concentrations of 0–250 μM was measured at 25 °C and a pH of 7.4, as described in Experimental Procedures. The concentrations of **3g** chosen were 0, 25, 187, and 2500 nM. Solid lines represent nonlinear regression fits to the data using the standard Michaelis-Menten kinetics, equation 2. (**B**) Dose-response profile of **3g** inhibition of thrombin in the presence of an exosite 1 ligand, hirudin peptide ([5F]-Hir-(54–65)-(SO_3_^−^)) at 25 °C in buffer of pH 7.4. (**C**) Dose-response profile of **3g** inhibition of thrombin in the presence of an exosite 2 ligand, unfractionated heparin. Solid lines represent sigmoidal dose-response fits using equation 1. (**D**) Comparison of *IC*_50_s of **3g** inhibition of recombinant human thrombins. The *IC*_50_s were measured using substrate hydrolysis assays, as shown in Fig. 3. The horizontal line is for comparison of change in *IC*_50_ of mutants from that of the wild-type recombinant human thrombin.

Thrombin possesses two electropositive anion-binding exosites that help regulate its catalytic activity (see also Fig. 1B)^19^,^20^. Exosite 1 is the site for interaction with fibrinogen, which propagates the clotting signal. It is also the site where hirudin and bivalirudin bind and inhibit thrombin. Exosite 2 is the site where unfractionated heparin (UFH), γ′-fibrinogen, and glycoprotein Ibα bind^19^,^20^,^42^,^43^,^44^,^45^. To assess whether **3g** interacts with one of these exosites, competition studies were performed. In the presence of fixed concentrations of two competing ligands, a hirudin peptide (HirP, [5F]-Hir-(54–65)-(SO_3_^−^)) for exosite 1 and UFH for exosite 2, the catalytic activity of thrombin was measured at varying concentrations of **3g** (Fig. 4B,C). As evident from the profiles, the presence of HirP marginally increased the *IC*_50_ of **3g** by 50%, whereas the presence of UFH reduced the potency of **3g** significantly by ~300% (Table 1). Further, comparison of the observed inhibition potency with that calculated on the basis of ideal competition using the Dixon-Webb relationship (see Experimental Procedures) suggests that **3g** competes almost ideally with UFH (Table 1), but not with HirP. Thus, **3g** binds in exosite 2 of thrombin and allosterically inhibits thrombin such that a maximal inhibition of only 50% is produced. The reason why HirP affects **3g** binding to a small extent is most probably coupling between the exosites, as has been documented in the literature^46^,^47^. Thus, **3g** is the first allosteric, partial inhibitor of thrombin. Although **3g** is a hydrophobic molecule with one sulfate group, it is not too surprising that it binds in the anion-binding exosite 2. A large number of sulfated small and large molecules have been developed to date and found to interact with exosite 2 of thrombin^27^,^28^,^36^,^41^ or exosite 2-like regions of other homologous coagulation factors^33^,^38^.

To further probe the **3g**–thrombin system, we studied a group of thrombin mutants containing replacement of either Arg or Lys present in exosite 2 to Ala. These site-directed mutants, studied earlier in depth in the Rezaie laboratory^48^,^49^, encompassed the majority of the electropositive residues present in exosite 2. In comparison to the recombinant wild-type thrombin control, the *IC*_50_s of **3g** inhibition of four mutants (K236A, K235A, R173A and K169A) increased by a small factor (<1.4-fold) (Fig. 4D, see also Supplementary Table S3 showing all parameters). Earlier studies on sulfated benzofurans have shown that disruption of a key interaction through Arg/Lys mutagenesis results in a considerable decrease in potency (~2–10-fold)^24^,^25^ and thus, these small changes suggest that K236, K235, R173 or K169 are most probably not singularly involved in **3g** recognition.

The inhibition potency of **3g** increased for five mutants (R233A, R165A, R126A, R101A, and R93A), of which R93A displayed a dramatic 2.3-fold effect. This is an unusual observation and opposes the expectation of a loss in potency. Yet, an analysis of thrombin crystal structure shows that a majority of these residues are located adjacent to hydrophobic sub-sites within/near exosite 2^50^,^51^. Thus, expansion of the hydrophobic sub-domain through Ala replacement of Arg/Lys may induce better recognition of **3g**’s aliphatic chain and/or aromatic rings resulting in enhancement of potency. This appears to be especially true for the R93A and R101A mutants, and possibly also for others. Earlier work on sulfated low molecular weight lignins has also shown the importance of hydrophobicity in recognition of exosite 2 and support the conclusion that **3g** binds in exosite 2 through primarily non-ionic forces^51^.

It is not clear why a specific electropositive amino acid residue was not identified as the primary site of **3g** recognition. It is possible that other Arg/Lys, such as R240, K107, K109, and K110 located within or adjacent to exosite 2, may be involved in interacting with the sulfate group of **3g**. It is also possible that the **3g** interacts with multiple Arg/Lys groups that are near neighbors, e.g., R240, K236, and K235, such that replacement of one does not adversely impact its affinity significantly. This possibility has previously been documented for the thrombin–low molecular weight lignin system and factor XIa–sulfated quinazolinone system, where single mutants showed much weaker changes in activity as compared to triple mutants^33^,^50^. Overall, this study of site-directed mutants supports the conclusion that **3g** utilizes hydrophobic forces in binding and that one or more Arg/Lys residue within or adjacent to exosite 2 may be the target of its sulfate group.

The study of recombinant thrombins also enables further confirmation of the partial inhibition phenomenon. Supplementary Table S3 lists the efficacies of each thrombin studied. The results show **3g** induces maximal inhibition efficacies in the range of 28 to 64%, which are comparable to that measured for sulfated coumarins inhibiting plasma thrombin. The retention of the partial inhibition phenomenon by recombinant thrombins shows that changes in the electrostatics of exosite 2 does not impact the intrinsic property of sulfated coumarins, especially **3g**.

### 3g Induces Conformational Allostery

In principle, allosterism may arise from conformational changes or dynamic changes introduced by the allosteric ligand. Conformational changes imply structural changes in the target macromolecule^52^,^53^, whereas dynamic changes imply alteration in the flexibility of the target macromolecule^54^,^55^,^56^. It is also possible that allostery arises from a combination of both types of changes. Considering that thrombin displays an ensemble of ground states (Fig. 1B)^11^,^21^,^57^, it is important to understand whether **3g** introduces conformational or dynamic allostery.

**Figure 5:**
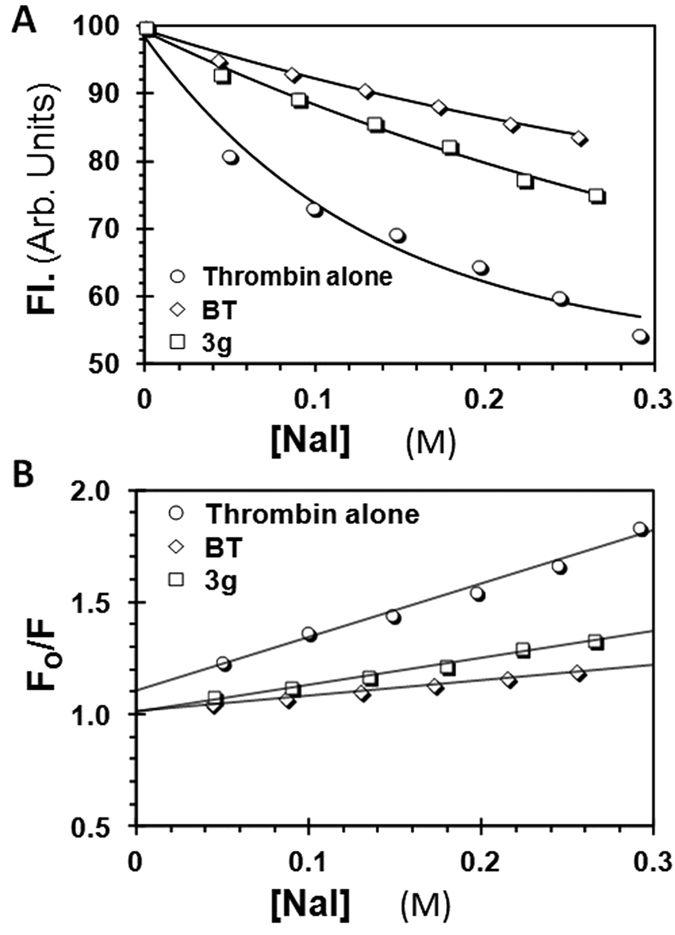
Conformational allostery induced by 3g in thrombin. (**A**) Change in *f* FPRCK-thrombin fluorescence with increasing concentrations of NaI in the presence of 1 μM **3g** and 2.5 μM **BT** in pH 7.4 buffer at 25 °C. Excitation and emission wavelengths were 490 nm and 525 nm, respectively. (**B**) Stern-Volmer analysis of the data presented above. Solid line represents linear fit to the data to calculate the slope, or sensitivity of the active site fluorophore to molecular collisions with iodide, at pH 7.4 and 25 °C.

To elucidate the nature of allostery, two orthogonal experiments were performed. First, fluorescence-quenching studies were performed on thrombin complexes with **3g** and a previously studied sulfated benzofuran trimer^28^ (**BT**), which displays inhibition efficacy >80%. Iodide anion is a well-established agent that is known to quench fluorescence through collisional interactions^58^. We reasoned that if **3g** induces less conformational changes in the protein in comparison to **BT**, then access of an active site fluorophore to the iodide anion should be different. Thus, quenching of fluoresceinylated thrombin, i.e., *f* FPRCK-thrombin, by iodide was studied in the presence and absence of **3g** and **BT**, respectively (Fig. 5A) and analyzed using Stern-Volmer relationships (Fig. 5B). The results revealed that the calculated slope of quenching for thrombin alone was 2.37 ± 0.14 M^−1^, which decreased to 1.19 ± 0.06 M^−1^ in the presence of **3g** (90% saturation) and to 0.69 ± 0.02 M^−1^ in the presence of **BT** (90% saturation). Thus, **3g** reduces iodide quenching of *f* FPRCK-thrombin by ~50%, while **BT** reduces by ~71%.

Although the above results indicate that **3g**, but not **BT**, induces partial conformational change in thrombin, a remote possibility of differential quenching by iodide anion is physical shielding by **3g**, but not **BT**. Both **3g** and **BT** bind in exosite 2^28^, which is too far away (~20 Å) from the site of the fluorescein label (thrombin’s S4 pocket). Further, this study shows that **3g** binding affinity correlates well with its *IC*_50_. If any part of **3g** structure was located at the S4 site, the affinity should have been lower. Thus, the results suggest that the observed efficacy (ΔY) of inhibition correlates with the ability of iodide anion to access to the active site fluorescein label, which is modulated by the allosteric inhibitor. Alternatively, **3g** induces a much smaller change in the orthosteric site in comparison to that of **BT**, which suggests a structural basis for its allosterism.

**Figure 6:**
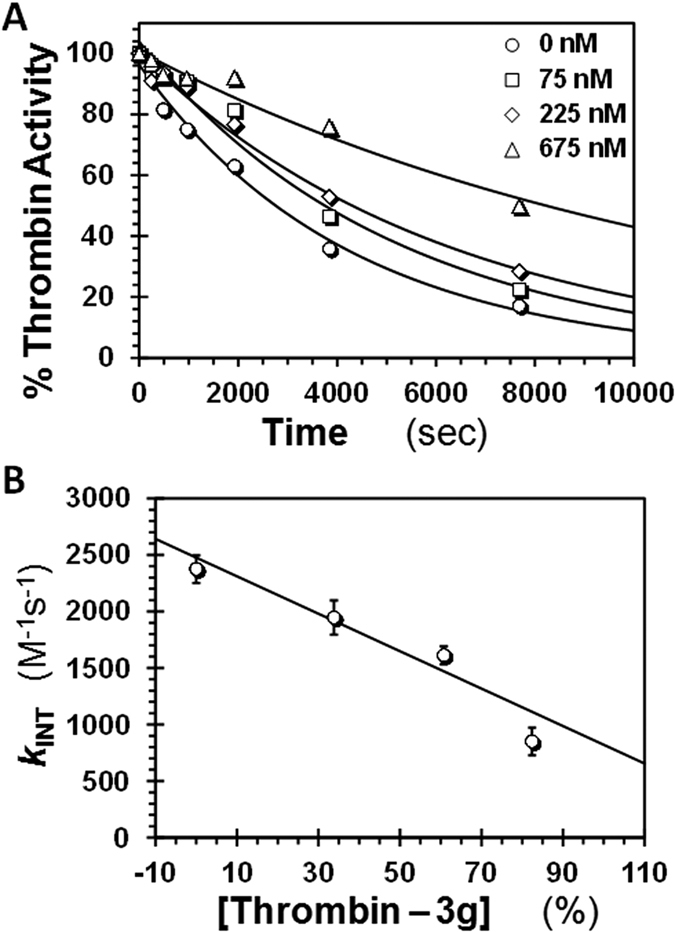
Structural changes induced in the active site of thrombin by 3g. (**A**) Residual thrombin activity as a function of time following incubation with excess antithrombin in the presence of fixed concentrations of **3g** (0–675 nM) in pH 7.2 buffer at 25 °C. Solid lines represent exponential fits to the data using equation 6 to calculate the pseudo first order rate constant *k*_OBS_. (**B**) Dependence of intrinsic second order rate constant of thrombin–antithrombin reaction (*k*_INT_), calculated from *k*_OBS_ (above) using equation 7, as a function of the percent saturation of thrombin at different levels of **3g** (i.e., [thrombin–**3g**]/[thrombin]). Solid line represents linear fit to the data to obtain the *k*_INT_ for thrombin–**3g** complex at pH 7.2 and 25 °C.

To further probe whether **3g** indeed induces changes in the orthosteric site, we studied the kinetics of the thrombin–antithrombin reaction in the presence of **3g** using a discontinuous spectrophotometric assay, as described earlier^59^,^60^. Figure 6A shows time profiles of thrombin inactivation at varying **3g** levels under pseudo-first order conditions, which were analyzed to derive the observed rate constant (*k*_OBS_). The intrinsic rate constant of antithrombin inhibition of thrombin, i.e., *k*_INT_, which is proportional to the concentration of antithrombin, was calculated from *k*_OBS_ using equation 6. A plot of *k*_INT_ *versus* percent saturation of thrombin with **3g** (Fig. 6) shows an expected linear relationship that displays an intercept of 2476 ± 228 M^−1^s^−1^ corresponding to the second order rate constant of antithrombin inhibition of thrombin alone in the absence of **3g**. More importantly, the *k*_INT_ at 100% saturation of thrombin, i.e., for thrombin–**3g** complex, was calculated to be 821 ± 198 M^−1^s^−1^. When similar experiments were performed for antithrombin–factor Xa system, **3g** did not induce any change in the second-order rate constant of inhibition (Supplementary Fig. S7). This demonstrates that the thrombin–**3g** complex retains significant proteolytic activity, in contrast to the nearly zero activity expected for a fully inhibited system. Thus, this suggests that **3g** induces an intermediate structural change in the active site of thrombin, which depresses the intrinsic reactivity of thrombin with antithrombin. If *k*_INT_ in the absence of **3g** is equated with 100% proteolytic activity, then these results imply that thrombin–**3g** complex is 33 ± 12% active, which corresponds reasonably well to the efficacy of **3g** inhibition of thrombin (ΔY ~49%). Therefore, **3g** inhibits thrombin by inducing a partial structural change in its active site.

### Significance

This work presents the first group of small molecules that induce partial allosteric inhibition in a monomeric protease. Mechanistically, this is very interesting and shows that it is possible to regulate monomeric proteases in a manner similar to multimeric proteins and receptors. Our work also shows that partial inhibition arises from a structural change in the catalytic site, just as partial agonism or antagonism is also associated with structural change at the distal site^2^,^17^. Thermodynamically, our work shows that it is not necessary for inhibition of monomeric proteases to exhibit a two-state equilibrium between native and fully inhibited states. As shown for thrombin, an intermediate state may exhibit sufficient stability to be exclusively populated when saturated by an appropriate ligand. The fact that such a phenomenon, i.e., partial allosterism of soluble, monomeric proteases, has not been documented in the literature highlights the challenge and value of this work.

Sulfated coumarins, especially **3g**, are able to bring about a limited conformational change in the active site of the thrombin, so as to still allow room for binding and cleavage of small substrates. Whether this phenomenon holds true for macromolecular substrates of thrombin^19^,^20^,^61^ such as fibrinogen, factor V, factor VIII, factor XI, factor XIII and protein C remains to be determined. If allosteric partial inhibition translates to one or more macromolecular substrates, it will offer significant advantages over other thrombin inhibitors that display 100% inhibition, e.g., dabigatran and argatroban. A simplistic prediction would be that partial allosteric inhibition of thrombin will enable a homeostatic state. For example, it can be expected to only depress fibrinogen cleavage, and not completely inhibit it, such that a balance between procoagulant and anticoagulant tendencies is achieved resulting in homeostasis^25^. Such a natural state is not achieved with orthosteric inhibitors used in the clinic today resulting in considerable bleeding^23^,^24^,^62^. Thus, the concept of partial allosteric inhibition in general, and specifically for thrombin, may have major implications for therapy.

Our work adds further support to conclusions derived in the literature that thrombin is a highly plastic enzyme^11^,^21^,^57^. Whether **3g** selects out one of the conformations from the ensemble present in its native state or induces a new form remains to be determined. Advanced stopped-flow kinetic studies, such as performed for thrombin – hirudin system^63^, may be able to clarify between the two possibilities. However, the discovery of partial allosterism for thrombin adds to the level of plasticity afforded by this key enzyme of the coagulation cascade. In fact, thrombin appears to exhibit properties well known for multimeric receptors.

In addition to the partial allosteric features, our work presents **3g** as the most potent small molecule exosite 2 inhibitor of thrombin discovered to date. The sulfated benzofurans developed earlier display potencies in the range of 0.7–200 μM^27^,^28^,^29^. Likewise, suramin, a sulfonated aromatic molecule, inhibits thrombin with 20–40 μM IC_50_^64^. Heparin hexasaccharide is not an inhibitor of thrombin, but binds with an affinity of ~8 μM^65^. The only molecules that target exosite 2 and inhibit thrombin with higher potency are the sulfated low molecular weight lignins (10–150 nM IC_50_)^30^,^36^,^39^, however, these are heterogeneous, oligomeric mixtures. Thus, **3g** could be classified as a good scaffold for the discovery of better partial allosteric regulators of hemostasis.

Overall, this initial discovery is extremely promising from the perspective of pursuing partial allosterism as a novel mechanism for regulators of monomeric proteases. It is possible that partial allosterism of monomeric proteases is a more general phenomenon and further effort will help identify ‘regulators’, rather than ‘inhibitors’, that lead to homeostasis.

## Experimental Methods

### Chemicals, Proteins, Substrates and Reagents

Anhydrous and chromatography solvents were from Sigma Aldrich (Milwaukee, WI) or Fisher Scientific (Pittsburgh, PA). Acetone-*d*_6_ and DMSO-*d*_6_ for NMR were from Sigma Aldrich (Milwaukee, WI) or Acros Organics (Morris Plains, NJ). Chemical reagents including BBr_3_, SO_3_•N(CH_3_)_3_, Cs_2_CO_3_, CuSO_4_•5H_2_O, sodium ascorbate, triethylamine (TEA), propargyl bromide, 1-bromo-3-chloropropane, 1-bromo-4-chlorobutane, sodium azide, and TBTA were from Sigma Aldrich (Milwaukee, WI), Fisher Scientific (Pittsburgh, PA), or Acros Organics (Morris Plains, NJ) and used as purchased. Coumarins were from Indofine Chemical Company Inc. (Hillsborough, NJ). All other chemicals were analytical reagent grade or higher and obtained from Sigma Aldrich (Milwaukee, WI) or Fisher Scientific (Pittsburgh, PA).

Human plasma proteases including thrombin, fluorescein-labeled (*f* FPRCK-)thrombin, antithrombin, factor Xa and factor XIa were from Haematologic Technologies (Essex Junction, VT). Recombinant thrombins were gifts from the Rezaie lab (St. Louis University). Spectrozyme TH (*H*-D-cyclohexylalanyl-Ala-Arg-*p*-nitroanilide) and Spectrozyme FXa (methoxycarbonyl-D-cyclohexylglycyl-Gly-Arg-*p*-nitroanilide) were from Sekisui Diagnostics (Lexington, MA). S-2366 (L-pyroGlu-Pro-Arg-*p*-nitroaniline•HCl) was from Diapharma (West Chester, OH). Bovine unfractionated heparin was from Sigma Aldrich and hirudin peptide (HirP, [5F]-Hir-(54–65)-(SO_3_^−^)) was from Anaspec (Fremont, CA).

### Enzyme Inhibition Studies

Chromogenic substrate hydrolysis assay was used to measure potency of inhibition, as previously reported^28^,^29^. The assay was performed using Flexstation III (Molecular Devices, Sunnyvale, CA) by monitoring substrate hydrolysis at 405 nm. For thrombin and its mutants, 180 μL of buffer (20 mM Tris-HCl, 100 mM NaCl, 2.5 mM CaCl_2_, 0.1% (polyethylene glycol) PEG 8000, pH 7.4) was added to each well followed by 5 μL of 240 nM thrombin and 10 μL of sulfated coumarin in DMSO to give 0.002 to 250 μM final concentration (or 10 μL of DMSO alone). After incubation for 10 min at 25 °C, 5 μL of 2 mM Spectrozyme TH was added to each well simultaneously and allowed to react for 60 seconds at 25 °C while simultaneously monitoring the reaction to obtain the rate of increase in A_405_. Identical experimental conditions were used for reactions in the presence of antithrombin, except for 100 nM of antithrombin added prior to the addition of the substrate.

For factor XIa inhibition studies, 85 μL of buffer (50 mM Tris-HCl, 150 mM NaCl, 0.1% PEG 8000, 0.02% Tween 80, pH 7.4) was added to each well, followed by 7 μL of 15.3 nM enzyme and 3 μL of sulfated coumarin (3.75–500 μM final concentration) and incubated for 10 min at 37 °C. After the incubation period, 5 μL of 6.9 mM S-2366 was added to each well simultaneously and allowed to react for 300 sec at 37 °C. For factor Xa assay, 180 μL of buffer (20 mM Tris-HCl, 100 mM NaCl, 2.5 mM CaCl_2_, 0.1% PEG 8000, 0.02% Tween 80, pH 7.4) was added to each well followed by 5 μL of 43.5 nM enzyme, 10 μL of sulfated coumarin (0.002–250 μM final concentration). After a 10 min incubation, 5 μL of 5 mM Spectrozyme FXa was added to each well simultaneously and allowed to react for 300 seconds at 37 °C. The slopes generated from each experiment were used to calculate the fractional residual activity (*Y*) at each concentration of the inhibitor, and analyzed using logistic equation 1, in which *Y*_M_ is the maximum efficacy, *Y*_0_ is minimum efficacy and HS is the Hill slope.


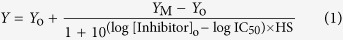


### Michaelis-Menten Kinetic Studies

Four concentrations (0, 25, 187, and 2500 nM) of **3g** were studied in triplicate where **3g** was incubated with thrombin for 10 min in buffer (20 mM Tris-HCl, 100 mM NaCl, 2.5 mM CaCl_2_, 0.1% PEG 8000, pH 7.4) at 25 °C. Spectrozyme TH at various concentrations (0–250 μM) was added at each of the four **3g** concentrations and the initial rate of hydrolysis was recorded from the increase in A_405_ as a function of time. This slope was fitted to the standard Michaelis-Menten equation to derive *V*_*MAX*_ and *K*_M_ of the reaction at each of the **3g** concentrations.


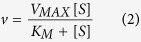


### Competition Studies with Exosites 1 and 2 Ligands

Thrombin inhibition by **3g** was examined in the presence of either HirP (exosite 1 ligand) or UFH (exosite 2 ligand). **3g** was prepared in DMSO (0.010–50 μM) and thrombin (4–8 nM) were incubated at 25 °C with HirP (0–187 nM) or UFH (0–22.8 μM) in buffer (20 mM Tris-HCl, 100 mM NaCl, 2.5 mM CaCl_2_, 0.1% PEG 8000, pH 7.4). After 10 min, thrombin activity was measured using Spectrozyme TH hydrolysis assay, as described above. The slopes obtained (in triplicate) were fitted using equation 1 to calculate *IC*_50,app_, ∆*Y*, and *HS*. The *IC*_50,app_ at each concentration of the competitor was also calculated using the Dixon-Webb relationship (equation 3) from the known affinities (*K*_comp_) of the competitor.


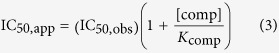


### Thermodynamic Affinity Studies

Affinity studies were performed in triplicate and conducted using quartz cuvettes. 180 μL of buffer (20 mM Tris-HCl, 100 mM NaCl, 2.5 mM CaCl_2_, 0.1% PEG 8000, pH 7.4) and 20 μL of 2 μM fluorescein-labeled (*f* FPRCK-) thrombin were added to the cuvette followed by titration with 1 μL additions of **3g**. Fluorescence intensities were monitored by excitation at 490 nm and emission at 525 nm using slit widths of 0.5 mm. The *K*_D_ of **3g**–thrombin complex was calculated using the quadratic binding equation (equation 4) in which ∆*F* is the change in fluorescence from the formation of the thrombin-ligand complex with each addition of **3g**. *F*_0_ is the initial fluorescence and ∆*F*_MAX_ represents the maximum observed change in fluorescence due to thrombin saturation ([Th]_0_). The binding was assumed to possess a stoichiometry of 1:1.


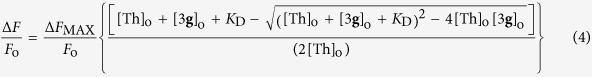


### Fluorescence Quenching Studies

The quenching studies performed were done using quartz cuvettes, as described previously^36^,^58^. 180 μL of buffer (20 mM Tris-HCl, 100 mM NaCl, 2.5 mM CaCl_2_, 0.1% PEG 8000, pH 7.4) and 20 μL of 2 μM fluorescein-labeled (*f* FPRCK-) human thrombin were added to the cuvette; fluorescent intensity readings were taken at the λ_EX_ of 490 nm and λ_EM_ of 525 nM at 25 °C. A solution of 10 M NaI was made using buffer as the solvent. For the readings where inhibitor was absent, 1 μL increments of 10 M NaI was added directly to the cuvette after initial readings of the buffer-thrombin solution. NaI was added six times, while recording changes in fluorescent intensity each time, giving a concentration range of 0–3.0 M in the cuvette. For the readings where inhibitor was present, after the initial buffer-thrombin read, 1 μL of 80 μM inhibitor solution was added to the cuvette and mixed. An intensity reading was taken and then the addition of 1 μL increments of 10 M NaI was again performed six times, taking reads between each addition. The experiments were performed in triplicate and averaged. Initial fluorescence (F_0_) was the intensity reading from either the buffer-thrombin solution in the absence of **3g** or following addition of 1 μL of **3g**. Linear regression was performed using Stern-Volmer relationship (equation 5), in which *F* is the intensity with quencher, [Q] is the concentration of quencher, and *K*_SV_ is the dynamic quenching constant given by the slope.


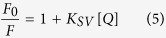


### Antithrombin Inactivation of Thrombin or Factor Xa in the Presence of Sulfated Coumarin

The effect of **3g** on the reaction of thrombin (or factor Xa) with antithrombin was studied under pseudo-first order conditions such that [AT]_0_ ≫ [T]_0_. A fixed concentration of 6 nM plasma α-thrombin in 20 mM Tris-HCl buffer, pH 7.2, containing 100 mM NaCl, 2.5 mM CaCl_2_ and 0.1% PEG 8000 at 25 °C was incubated for 128 min with final concentrations of 0, 75, 225, or 675 nM of **3g** following which a fixed concentration of 100 nM of antithrombin was added and the reaction allowed to proceed. Similarly, 5 nM of plasma factor Xa in 20 mM Tris-HCl buffer, pH 7.2, containing 100 mM NaCl, 2.5 mM CaCl_2_ and 0.1% PEG 8000 at 25 °C was incubated for 210 min with final concentrations of 0 or 220 nM of **3g** following addition of a fixed concentration of 100 nM of antithrombin was added and the reaction allowed to proceed. At a defined time point, a small aliquot of Spectrozyme TH was added to a final concentration of 50 μM for thrombin and Spectrozyme Xa a final concentration of 150 μM for factor Xa. The initial rate of hydrolysis of the Spectrozymes were monitored from the linear increase in A_405_. The fractional residual enzyme activity at each time point was calculated from the slope, i.e., thrombin activity, measured at the start of the experiment and fitted by the standard exponential decay equation 6 to calculate the observed pseudo-first order rate constant, *k*_OBS_, at each concentration of **3g**. The intrinsic second-order rate constant of antithrombin inhibition of thrombin (*k*_INT_) was calculated using equation 7 and plotted against the concentration of thrombin–**3g** complex, obtained from quadratic equation 8, to derive the *k*_INT_ of antithrombin inhibition of thrombin–**3g** complex.


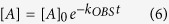



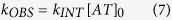



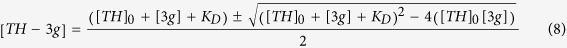


## Additional Information

**How to cite this article**: Verespy III, S. *et al*. Allosteric Partial Inhibition of Monomeric Proteases. Sulfated Coumarins Induce Regulation, not just Inhibition, of Thrombin. *Sci. Rep.* **6**, 24043; doi: 10.1038/srep24043 (2016).

## Supplementary Material

Supplementary Information

## Figures and Tables

**Table 1:** Thrombin inhibition by 3g in the presence of HirP and UFH^a^.

	*IC*_50,app_ (μM)^b^	Δ*Y*(%)^b^	*HS*	*IC*_50,predicted_ (μM)^c^
[HirP] (nM)				
0	0.22 ± 0.02	52 ± 2	3.3	0.24
25	0.33 ± 0.02	58 ± 1	2.4	0.45
187	0.33 ± 0.06	59 ± 6	2.0	0.67
[UFH] (μM)				
0	0.17 ± 0.02	56 ± 3	1.3	0.17
11.4	0.29 ± 0.03	66 ± 5	1.7	0.29
22.8	0.58 ± 0.03	59 ± 3	3.7	0.45

^a^Inhibition was measured at pH 7.4 and 25 °C using chromogenic substrate hydrolysis assay and analyzed using logistic equation 1 to obtain *IC*_50,app_, ΔY and *HS*.

^b^Errors represent ± 1 SE obtained from non-linear regression of the inhibition profile.

^c^Calculated using Dixon-Webb relationship (equation 3).
