# Supplementary material for: Allosteric Partial Inhibition of Monomeric Proteases. Sulfated Coumarins Induce Regulation, not just Inhibition, of Thrombin
Source: Sci Rep. 2016 Apr 7;6:24043. doi: 10.1038/srep24043 (PMC4823711; doi:10.1038/srep24043)
Supplement: Supplementary Information [file srep24043-s1.pdf]

Supporting Information

Allosteric Partial Inhibition of Monomeric Proteases. Sulfated Coumarins Induce Regulation, not just Inhibition, of Thrombin

Stephen Verespy III, Akul Y. Mehta, Daniel Afosah, Rami A. Al-Horani, and Umesh R. Desai*

Department of Medicinal Chemistry and Institute for Structural Biology, Drug Discovery and Development, Virginia Commonwealth University, Richmond, Virginia 23219

*Address for correspondence: Dr. Umesh R. Desai, 800 E. Leigh Street, Suite 212, Richmond, VA 23219. Ph (804) 828 7328; Fax (804) 827 3664; urdesai@vcu.edu.

Table of Contents

1. General Synthetic Procedure Pg. 2-4
2. Table S1: Inhibition of Thrombin, Factor Xa, and Factor XIa by Sulfated Coumarins Pg. 5
3. Table S2: Michaelis-Menten Kinetics of Spectrozyme TH Hydrolysis by Thrombin in the Presence of 3g Pg. 6
4. Table S3: Inhibition of Recombinant Wild Type Thrombin and Thrombin Mutants Pg. 6
5. Figure S1: General Sulfation Scheme Pg. 7
6. Figure S2: General Demethylation and Sulfation Scheme Pg. 7
7. Figure S3: Dimer Synthesis Scheme Pg. 8
8. Figure S4: ROESY of **3f3** Pg. 9
9. Figure S5: ROESY of **3g4**/**3f4** Pg. 10
10. Figure S6: ROESY of **3g** Pg. 11
11. Spectra Data S1: Spectral Data – Intermediates Pg. 12-13
12. Spectra Data S2: Spectral Data – Sulfated Products Pg. 14-19
13. Figure S7: Antithrombin-FXa Inactivation Study Pg. 19

General Procedure for O-Demethylation — *O*-methylated analogs were suspended in dry DCM and subjected to 1.5 eq. per methoxy group of 1.0 M BBr_3 solution at -78 ˚C under N_2 atmosphere. The solutions were stirred for 5 h, allowed to reach RT, following which the solutions were re-cooled to 0 ˚C and water (or 1:1 water/methanol) were slowly added with stirring to quench the reaction. The biphasic solution was then condensed under vacuum and 20 mL ethyl acetate and 25 mL saturated ammonium chloride solutions were sequentially added to the mixture. The aqueous phase was washed twice with 10-20 mL ethyl acetate and the organic layers were pooled, washed with brine, dried over an. Na_2SO_4 and then condensed under vacuum, followed by purification via flash chromatography (0-70% ethyl acetate/hexanes). Fractions that contained the demethylated products were pooled to yield white or yellow solids in 87 – 98% yields in purity greater than 95%, confirmed by UPLC. Products of **2w-2y** were then directly subjected to sulfation. (**2w-2y**, **3a1-3g1**, see Figure 1, Supplementary Figures S2 & S3, and other Supplementary Materials)

General Procedure for Dimer Synthesis — To prepare dimeric C-SAMs **3a-3g** (Table 1), we employed copper (I)-catalyzed azide-alkyne cycloaddition (CuAAC) reaction, as described earlier.[34] Supplementary Figure S3 shows the preparation of the alkylazide scaffold. Scaffolds **3a1-3g1** containing two phenolic -OH groups (at the 7-hydroxy position and 3-[4'-hydroxyphenyl]) were treated with 0.9 – 1.0 molar eq. of 1-bromo-3-chloropropane/1-bromo-4-chlorobutane and 0.5 eq. of Cs_2CO_3 in DMF for 24 h at room temperature. Upon completion of the reaction, an acidic work up was followed by extraction with ethyl acetate and standard chromatographic purification to give the intermediates **3a2-3g2** in 66–83% yields. The resulting alkylchloride intermediate was dissolved in 3:1 DMF/ACN, transferred to a 10 mL microwave vessel containing 1.5 eq. of sodium azide. The vessel was sealed, placed in the microwave reactor, and heated for 5 h at 70 ºC. Upon cooling to RT, the solution was mixed with 10-20 mL ethyl acetate and 10 mL 1.0 M HCl and the organic layer separated and worked up to yield corresponding the alkyl azide derivatives **3a3-3g3** in 90 – 97% yields after flash chromatography and >94% purity confirmed by UPLC. The products were characterized using ^{1}H-NMR, ROESY, and IR (see Supplementary Figure S3-S4 and Spectra Data S1).

Supplementary Figure S3 also shows the preparation of the propargyl scaffold. To produce the alkyne reactant, the analogs displayed two OH groups as stated above. **3a-3g** and 0.9-1.0 eq. of 80% propargyl bromide solution were added to a stirring solution of 0.5 eq. of Cs_2CO_3 in DMF and continued to stir at room temperature for 24 h. Upon completion, 20 mL of ethyl acetate and 15 mL of 1.0 M HCl were added to the reaction and the organic layer was separated and worked up to yield the propargyl derivatives **3a4-3g4** in 58-89% yields after flash chromatography and >95% purity confirmed by UPLC. The structure and propargyl positioning were confirmed by ^{1}H-NMR and ROESY (see Supplementary Figure S3 and S5 and Spectra Data S1).

The intermediates **3a3-3g3** and **3a4-3g4** were coupled together, respectively under CuAAC condition. 1.1 eq of azide intermediate and 1.0 eq of alkyne intermediate were combined and stirred in DMF with 7 mol% of sodium ascorbate solution in water and 7 mol% 1:1 $CuSO_4$•$5H_2O$: tris-(benzyltriazolylmethyl)amine (TBTA) in 55% DMSO for 24 h at RT. The reaction was then quenched with 15 mL of chilled water to form precipitants, then filtered, and washed 20 mL of water twice. The precipitant collected was then dissolved in 25 mL ethyl acetate, which was washed with brine, dried over Na_2SO_4, and purified via flash chromatography giving the coupled products **3a5-3g5** in 72-94% yields with >95% purity, confirmed by UPLC. Structural identification was performed by ^{1}H-NMR (see Supplementary Figure S3 and Spectra Data S1).

General Procedure for Sulfation of Coumarin Derivatives — Derivatives composed of the free phenolic-OH were then subject to microwave assisted sulfation, Supplementary Figure S1-S3 shows the preparation of sulfation of **1a-1d**, **2a-2y**, and **3a-3g**. The compounds were added to a microwave reaction vessel with 6 eq of SO_3•$N(CH_3)_3$ per OH, 10 eq of TEA per OH, and 2-5 mL of ACN (or ACN/DMF). The vessel was then placed in the microwave reactor for 1 h per OH at 90-100 ºC. The solution and vessel were washed out with 10 mL of DCM and purified by flash chromatography (0-30% methanol/DCM), affording the sulfate containing the trimethylamine counter ion. Cation exchange chromatography was performed on the purified compounds and lyophilized to give the sodium sulfate final products in high

yields (82-99%) with >95% purity confirmed by UPLC-ESI-MS, along with structural identification by ^{1}H-NMR, ^{13}C-NMR, and ROESY (see Supplementary Figure S6 and Spectra Data S2).

Table S1. Inhibition of thrombin, factor Xa and factor XIa by sulfated coumarins.[a]

	**Thrombin**			**Factor Xa**			**Factor XIa**		
	***IC*$_{50}$ (μM)**[b]	**Δ*Y* (%)**[b]	***HS***	***IC*$_{50}$ (μM)**[b]	**Δ*Y* (%)**[b]	***HS***	***IC*$_{50}$ (μM)**[b]	**Δ*Y* (%)**[b]	***HS***
**2e**	58 ± 7	66 ± 6	2.7	62 ± 20	60 ± 20	2.9	220 ± 12	86 ± 7	4.1
**2k**	7.2 ± 2	22 ± 4	2.9	65 ± 23	54 ± 14	1.9	137 ± 7	83 ± 6	2.8
**2o**	NI[c]	-[d]	-[d]	53 ± 28	91 ± 26	1.2	NI[c]	-[d]	-[d]
**2p**	35 ± 1	35 ± 1	5.3	NI[c]	-[d]	-[d]	NI	-	-
**3a**	0.5 ± 0.1	73 ± 6	1.3	56 ± 29	92 ± 23	1.1	51 ± 2	88 ± 2	3.5
**3b**	1.0 ± 0.1	58 ± 2	2.6	13 ± 3	67 ± 14	6.7	59 ± 3	95 ± 3	4.3
**3c**	7.8 ± 1	60 ± 6	1.2	NI	-	-	78 ± 3	100 ± 3	4.3
**3d**	11 ± 2	34 ± 3	9.2	NI	-	-	91 ± 6	97 ± 5	3.4
**3e**	20 ± 3	52 ± 7	3.9	NI	-	-	97 ± 4	98 ± 4	4.0
**3f**	8 ± 3	36 ± 9	9.8	NI	-	-	73 ± 2	97 ± 2	3.9
**3g**	0.2 ± 0.1	47 ± 3	1.2	163 ± 2	83 ± 3	1.8	31 ± 0	99 ± 3	3.5
**3h**	NI	-	-	NI	-	-	NI	-	-

[a] Inhibition of each coagulation factor was measured at pH 7.4 and 25 °C using chromogenic substrate hydrolysis assay and analyzed using sigmoidal dose-response equation 1 to obtain IC_{50}, ΔY, and HS.

[b]Errors represent ± 1 SE obtained from non-linear regression of inhibition profile.

[c]Did not inhibit the protease under the experimental conditions below a concentration of 250 μM.

[d]Not applicable

Table S2. Michaelis-Menten kinetics of Spectrozyme TH hydrolysis by thrombin in the presence of **3g**.[a]

[**3g**] (nM)	K_M (μM)[b]	V_{MAX} (mAU/min)[b]
0	9.8 ± 1.0	57.5 ± 1.3
25	6.9 ± 1.0	52.4 ± 1.5
187	8.5 ± 1.0	45.3 ± 1.1
2500	7.2 ± 1.0	31.0 ± 0.9

[a] Substrate hydrolysis was measured at pH 7.4 and 25 °C using chromogenic substrate hydrolysis assay and analyzed using standard Michaelis – Menten equation 2 to obtain K_M and V_{MAX} values.

[b]Errors represent ± 1 SE obtained from non-linear regression of inhibition profile.

Table S3. Inhibition of recombinant wild type thrombin and thrombin mutants.[a]

	$IC_{50,app}$ (μM)[b]	ΔY (%)[b]	***HS***
wtTH	0.413 ± 0.05	39 ± 3	4.0
K236A	0.525 ± 0.07	64 ± 4	2.6
K235A	0.471 ± 0.08	33 ± 3	1.9
R233A	0.359 ± 0.07	33 ± 4	2.0
R175A	0.409 ± 0.08	42 ± 5	2.2
R173A	0.522 ± 0.09	36 ± 3	2.0
K169A	0.494 ± 0.05	38 ± 3	7.6
R165A	0.301 ± 0.02	28 ± 2	4.2
R126A	0.308 ± 0.03	41 ± 2	3.2
R101A	0.255 ± 0.05	35 ± 4	2.5
R97A	0.380 ± 0.07	41 ± 4	2.4
R93A	0.154 ± 0.02	51 ± 2	1.3

[a] Inhibition was measured at pH 7.4 and 25 °C using chromogenic substrate hydrolysis assay and analyzed using logistic equation 1 to obtain $IC_{50,app}$, ΔY and *HS* values.

[b]Errors represent ± 1 SE obtained from non-linear regression of the inhibition profile.

Figure S1 – General Sulfation

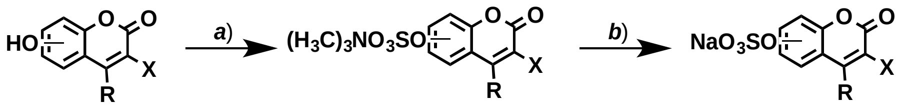

Figure S1: General scheme of sulfation for monomeric, free-OH C-SAM synthesis (**1a-1d**, **2a-2v**). *a*) SO_3•$N(CH_3)_3$, TEA, ACN, MW, 100ºC, 1-3h; *b*) Cation exchange chromatography. R- and X-groups correspond to the variety of substituents shown in Figure 1 of text.

Figure S2 – Demethylation and Sulfation

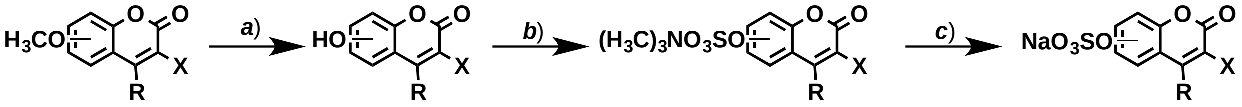

Figure S2: General scheme for a C-SAM *O*-demethylation and sulfation (**2w-2y**). *a*) 1.0M BBr_3, dry DCM, -78 ºC, 5h; *b*) SO_3•$N(CH_3)_3$, TEA, ACN, MW, 100ºC, 1-3h; *c*) Cation exchange chromatography. R- and X-groups signify substituents found in Figure 1.

Figure S3 – Dimer Synthesis and Sulfation

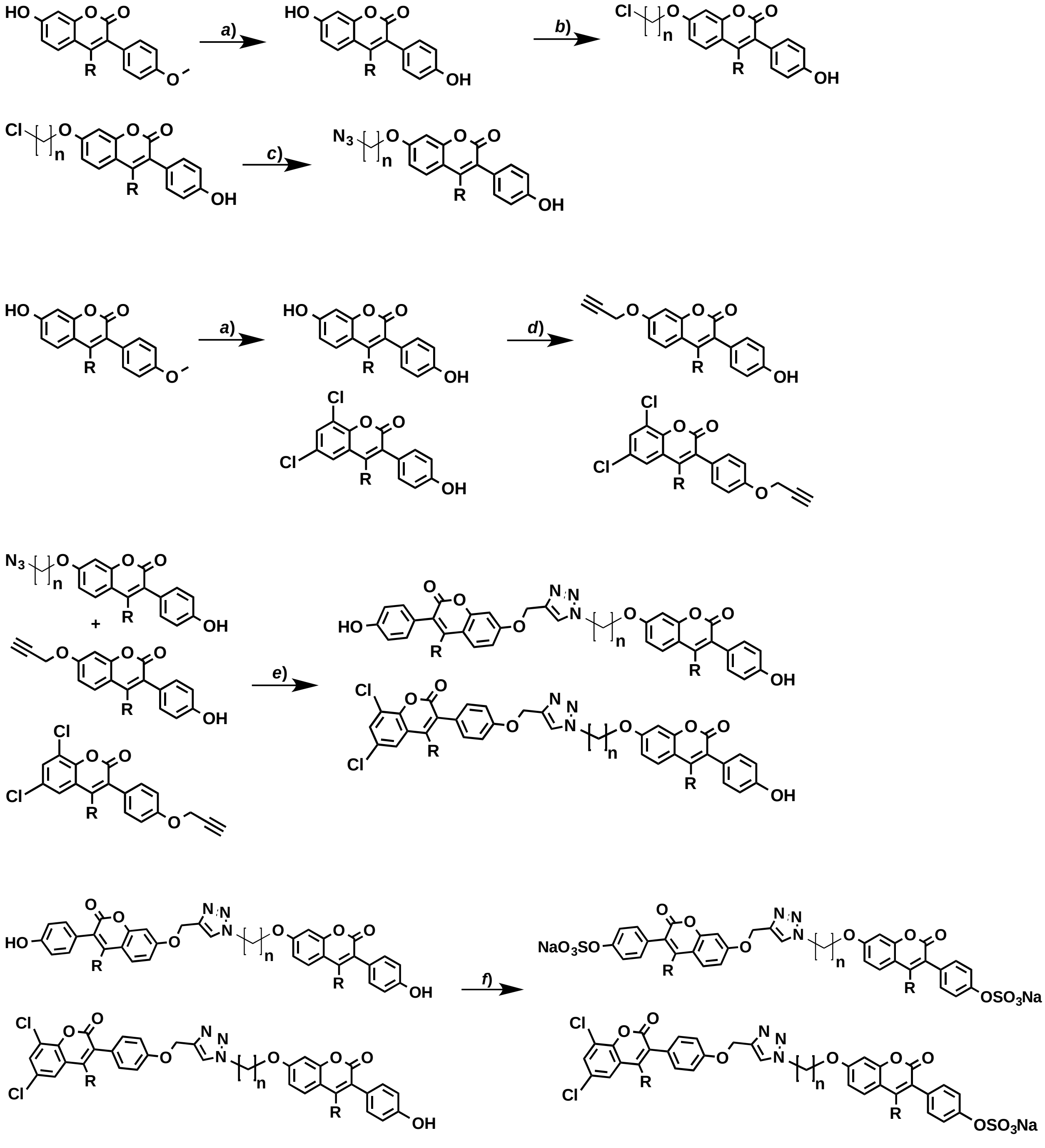

Figure S3: Stepwise procedure for intermediate and dimer synthesis. (a) 1M BBr_3, DCM, -78 ºC, 5h; (b) 1-bromo-3-chloropropane / 1-bromo-4-chlorobutane, Cs2CO_3, DMF, rt, overnight; (c) sodium azide, DMF, MW, 70 ºC, 5h; (d) propargyl bromide, Cs2CO_3, DMF, rt, overnight; (e)sodium ascorbate,$CuSO_4$ • $5H_2O$/TBTA, DMF/DMSO, rt, overnight; (f) SO_3•$N(CH_3)_3$, TEA, ACN, MW, 100 ºC, 1-3h. R- and n-groups correspond to Figure 2.

Figure S4 – ROESY of 3e4/3f4

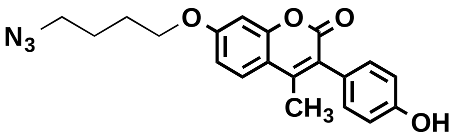

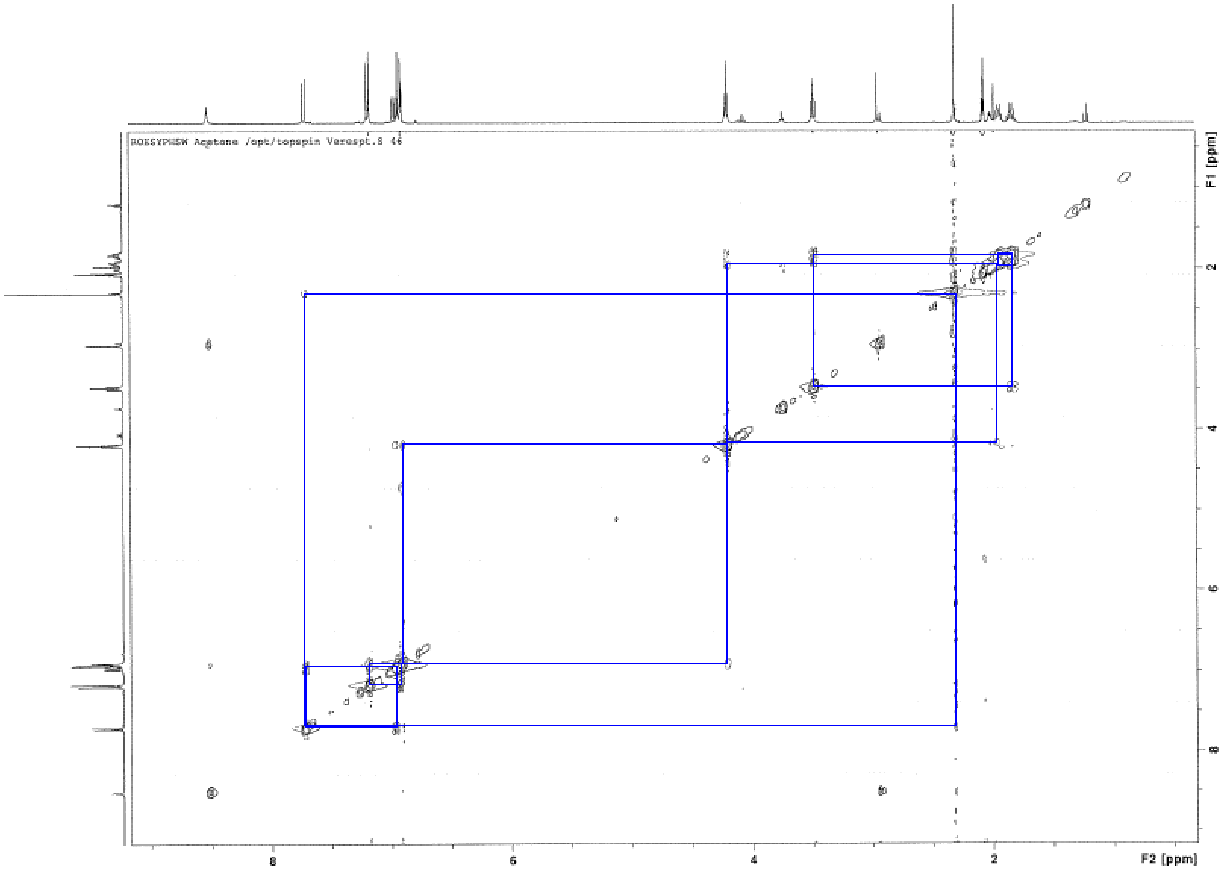

Figure S5 – ROESY of 3e4/3f4

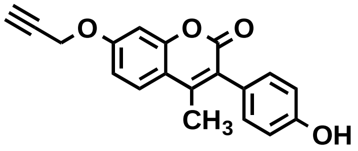

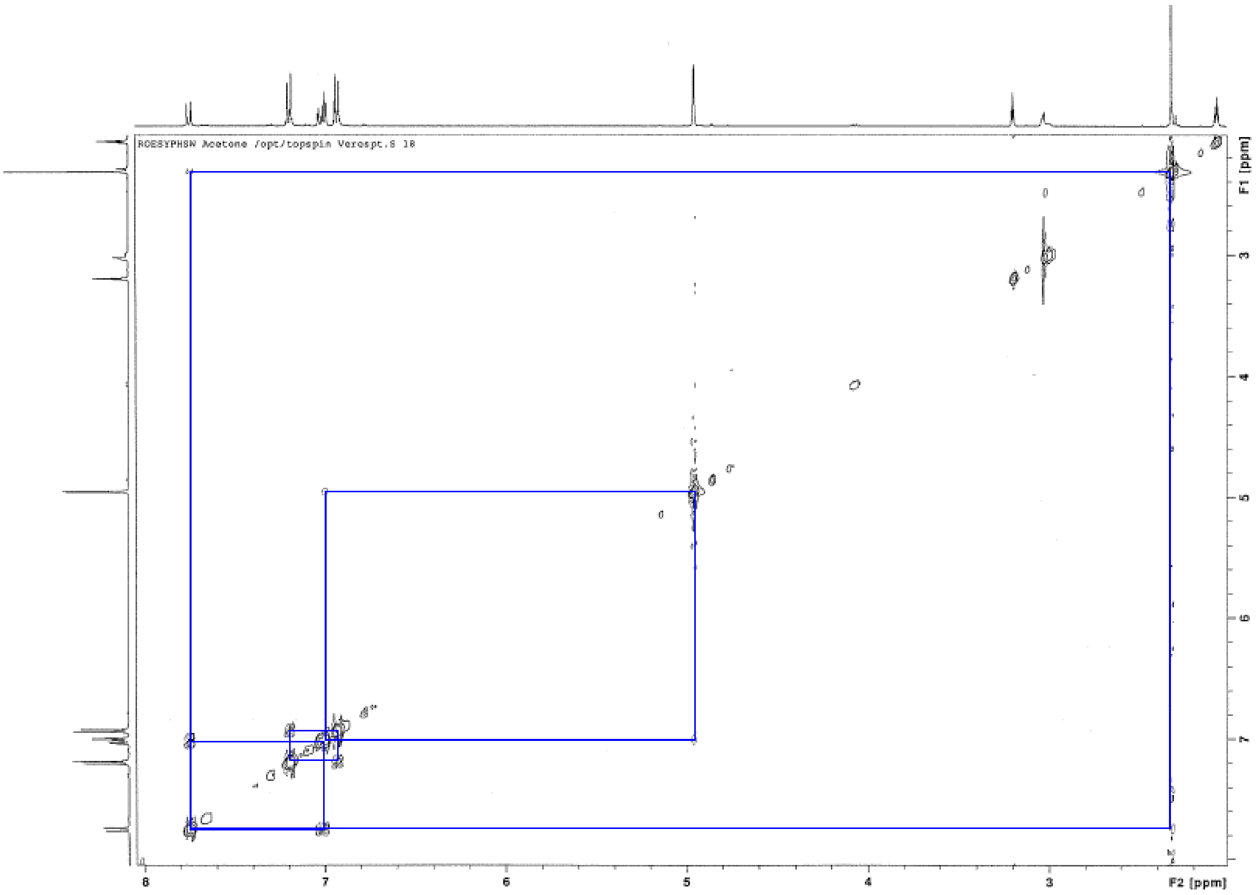

Figure S6 – ROESY of 3g

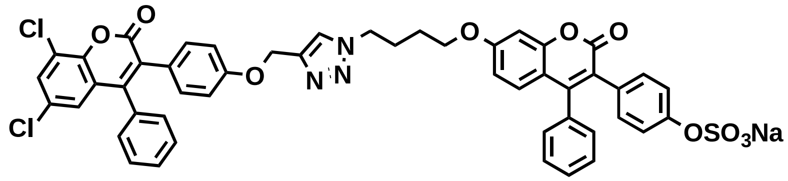

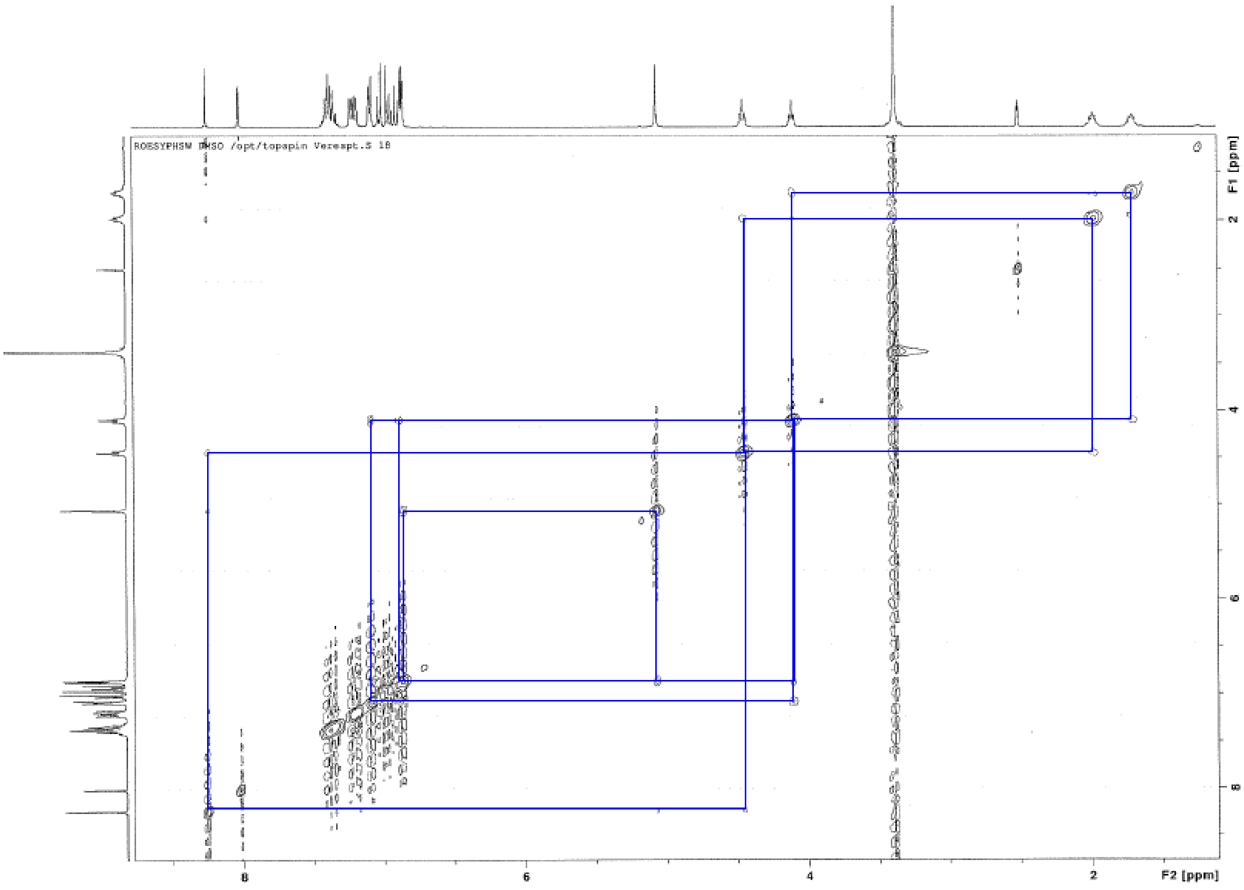

Spectra Data S1 –Spectral Data – Intermediates

3a1/3b1/3g1 – ^{1}H NMR (Acetone-d_6 400 MHz): 7.36 (m, 3H), 7.17 (dd, *J* = 8 Hz, 1.8 Hz, 2H), 6.91 (d, *J* = 8 Hz, 2H), 6.88 (d, *J* = 8 Hz, 1H), 6.82 (d, *J* = 4 Hz, 1H), 6.74 (dd, *J* = 8 Hz, 2.36 Hz, 1H), 6.54 (d, *J* = 8 Hz, 2H).

3c1/3d1 – ^{1}H NMR (Acetone-d_6 400 MHz): 7.81 (s, 1H), 7.50 (d, *J* = 8 Hz, 2H), 7.44 (d, *J* = 8 Hz, 1H), 6.77 (d, *J* = 8 Hz, 2H), 6.74 (dd, *J* = 8 Hz, 2.32 Hz, 1H), 6.65 (d, *J* = 8 Hz, 1H).

3e1/3f1 – ^{1}H NMR (Acetone-d_6 400 MHz): 7.71 (d, *J* = 8 Hz, 1H), 7.21 (d, *J* = 8 Hz, 2H), 6.95 (d, *J* = 8 Hz, 2H), 6.91 (d, *J* = 8 Hz, 1H), 6.81 (d, *J* = 8 Hz, 1H), 2.33 (s, 3H).

3a3 – ^{1}H NMR (Acetone-d_6 400 MHz): 7.23 (m, 3H), 7.10 (dd, *J* = 8 Hz, 2.48 Hz, 2H), 6.93 (m, 2H), 6.89 (d, *J* = 8 Hz, 2H), 6.80 (dd, *J* = 8 Hz, 2.54 Hz, 1H), 6.52 (d, *J* = 8 Hz, 2H), 4.28 (t, *J* = 8 Hz, 2H), 4.02 (t, *J* = 8 Hz, 2H), 2.21 (quint, *J* = 8 Hz, 2H). IR (neat): 2102 cm^{-1}.

3b3/3g3 – ^{1}H NMR (Acetone-d_6 400 MHz): 7.24 (m, 3H), 7.09 (dd, *J* = 8 Hz, 1.84 Hz, 2H), 6.91 (m, 3H), 6.73 (dd, *J* = 8 Hz, 2.48 Hz, 1H), 6.50 (d, *J* = 8 Hz, 2H) 4.08 (t, *J* = 8 Hz, 2H), 3.34 (t, *J* = 8 Hz, 2H), 1.81 (quint, *J* = 8 Hz, 2H), 1.72 (quint, *J* = 8 Hz, 2H). IR (neat): 2098 cm^{-1}.

3c3 – ^{1}H NMR (Acetone-d_6 400 MHz): 7.98 (s, 1H), 7.69 (d, *J* = 8 Hz, 1H), 7.61 (d, *J* = 8 Hz, 2H), 7.05 (s, 1H), 7.01 (dd, *J* = 8 Hz, 2.52 Hz, 1H), 6.88 (d, *J* = 8 Hz, 2H), 4.32 (t, *J* = 8 Hz, 2H), 3.98 (t, *J* = 8 Hz, 2H), 2.34 (quint, *J* = 8 Hz, 2H). IR (neat): 2119 cm^{-1}.

3d3 – ^{1}H NMR (Acetone-d_6 400 MHz): 7.96 (s, 1H), 7.67 (d, *J* = 8 Hz, 1H), 7.58 (d, *J* = 8 Hz, 2H), 7.02 (s, 1H), 6.99 (dd, *J* = 8 Hz, 2.36 Hz, 1H), 6.84 (d, *J* = 8 Hz, 2H), 4.15 (t, *J* = 8 Hz, 2H), 3.45 (t, *J* = 8 Hz, 2H), 1.86 (quint, *J* = 8 Hz, 2H), 1.75 (quint, *J* = 8 Hz, 2H). IR (neat): 2107 cm^{-1}.

3e3 – ^{1}H NMR (Acetone-d_6 400 MHz): 7.61 (d, *J* = 8 Hz, 1H), 7.05 (d, *J* = 8 Hz, 2H), 6.87 (dd, *J* = 8 Hz, 2.52 Hz, 1H), 6.80 (d, *J* = 4 Hz, 1H), 6.79 (d, *J* = 8 Hz, 2H), 4.19 (t, *J* = 8 Hz, 2H), 3.73 (t, *J* = 8 Hz, 2H), 2.31 (s, 3H), 2.21 (quint, *J* = 8 Hz, 2H), 2.18 (s, 3H). IR (neat): 2112 cm^{-1}.

3f3 – ^{1}H NMR (Acetone-d_6 400 MHz): 7.73 (d, *J* = 8 Hz, 1H), 7.20 (d, *J* = 8 Hz, 2H), 6.98 (dd, *J* = 8 Hz, 2.52 Hz, 1H), 6.93 (d, *J* = 8 Hz, 2H), 6.91 (d, *J* = 8 Hz, 1H), 4.22 (t, *J* = 8 Hz, 2H), 3.50 (t, *J* = 8 Hz, 2H), 2.31 (s, 3H), 1.98 (quint, *J* = 8 Hz, 2H), 1.87 (quint, *J* = 8 Hz, 2H). IR (neat): 2130 cm^{-1}.

3a4/3b4 – ^{1}H NMR (Acetone-d_6 400 MHz): 7.23 (m, 3H), 7.10 (m, 2H), 6.95 (m, 2H), 6.88 (d, *J* = 8 Hz, 2H), 6.78 (dd, *J* = 8 Hz, 2.52 Hz, 1H), 6.51 (d, *J* = 8 Hz, 2H), 4.81 (s, 2H), 3.03 (s, 1H).

3c4/3d4 – ^{1}H NMR (Acetone-d_6 400 MHz): 7.86 (s, 1H), 7.54 (m, 3H), 6.88 (d, *J* = 8 Hz, 2H), 6.78 (d, *J* = 8 Hz, 2H), 4.82 (s, 2H), 3.04 (s, 1H).

3e4/3f4 – ^{1}H NMR (Acetone-d_6 400 MHz): 7.61 (d, *J* = 8 Hz, 1H), 7.05 (d, *J* = 8 Hz, 2H), 6.89 (dd, *J* = 8 Hz, 2.52 Hz, 1H), 6.80 (d *J* = 8 Hz, 1H), 6.78 (d, *J* = 8 Hz, 2H), 4.82 (s, 2H), 3.04 (s, 1H), 2.30 (s, 3H).

3g4 – ^{1}H NMR (Acetone-d_6 400 MHz): 7.64 (d, *J* = 4 Hz, 1H), 7.29 (m, 3H), 7.17 (dd, *J* = 8 Hz, 1.92 Hz, 2H), 7.02 (d, *J* = 8 Hz, 2H), 6.90 (d, *J* = 2.44 Hz, 1H), 6.65 (d, *J* = 8 Hz, 2H), 4.79 (s, 2H), 3.09 (s, 1H).

3a5 – ^{1}H NMR (Acetone-d_6 400 MHz): 8.37 (s, 1H), 7.39 (m, 6H), 7.29 (s, 1H), 7.22 (m, 4H), 7.11 (s, 1H), 7.04 (m, 4H), 6.97 (m, 7H), 6.88 (dd, *J* = 8 Hz, 2.4 Hz, 1H), 5.29 (s, 2H), 4.61 (t, 2H), 4.16 (t, 2H), 2.35 (quint, *J* = 8 Hz, 2H).

3b5 – ^{1}H NMR (Acetone-d_6 400 MHz):): 8.35 (s, 1H), 7.37 (m, 6H), 7.26 (s, 1H), 7.15 (m, 4H), 7.08 (s, 1H), 7.04 (m, 4H), 6.91 (m, 4H), 6.87 (s, 1H), 6.54 (dd, *J* = 8 Hz, 2.36 Hz, 1H), 5.29 (s, 2H), 4.50 (t, *J* = 8 Hz, 2H), 4.15 (t, *J* = 8 Hz, 2H), 2.05 (quint, *J* = 8 Hz, 2H), 1.78 (quint, *J* = 8 Hz, 2H).

3c5 – ^{1}H NMR (Acetone-d_6 400 MHz): 8.35 (s, 1H), 8.08 (s, 2H), 7.68 (m, 2H), 7.58 (d, *J* = 8 Hz, 4H), 7.18 (s, 1H), 7.05 (m, 3H), 6.84 (d, *J* = 8 Hz, 2H), 5.29 (s, 2H), 4.61 (t, *J* = 8 Hz, 2H), 4.14 (t, *J* = 8 Hz, 2H) 2.38 (quint, *J* = 8 Hz, 2H).

3d5 – ^{1}H NMR (Acetone-d_6 400 MHz): 8.33 (s, 1H), 8.07 (s, 2H), 7.68 (m, 2H), 7.58 (d, *J* = 8 Hz, 4H), 7.18 (s, 1H), 7.04 (m, 2H), 6.85 (d, *J* = 8 Hz, 4H), 5.29 (s, 2H), 4.50 (t, *J* = 8 Hz, 2H), 4.13 (t, *J* = 8 Hz, 2H), 2.06 (quint, *J* = 8 Hz, 2H), 1.77 (quint, *J* = 8 Hz, 2H).

3e5 – ^{1}H NMR (Acetone-d_6 400 MHz): 8.37 (s, 1H), 8.00 (s, 2H), 7.57 (t, *J* = 8 Hz, 2H), 7.04 (s, 1H), 7.02 (d, *J* = 8 Hz, 4H), 6.92 (dd, *J* = 8 Hz, 2.52 Hz, 1H), 6.89 (m, 2H), 6.74 (d, *J* = 8 Hz, 4H), 5.21 (s, 2H), 4.58 (t, *J* = 8 Hz, 2H), 4.08 (t, *J* = 8 Hz, 2H), 2.35 (quint, *J* = 8 Hz, 2H), 2.16 (s, 6H).

3f5 – ^{1}H NMR (Acetone-d_6 400 MHz): 8.33 (s, 1H), 7.96 (s, 2H), 7.75 (t, *J* = 8 Hz, 2H), 7.17 (s, 1H), 7.11 (d, *J* = 8 Hz, 4H), 7.07 (dd, *J* = 8 Hz, 2.52 Hz, 1H), 6.99 (m, 2H), 6.84 (d, *J* = 8 Hz, 4H), 5.30 (s, 2H), 4.51 (t, *J* = 8 Hz, 2H), 4.14 (t, *J* = 8 Hz, 2H), 2.25 (s, 6H), 2.04 (quint, *J* = 8 Hz, 2H), 1.77 (quint, *J* = 8 Hz, 2H).

3h – ^{1}H NMR (Acetone-d_6 400 MHz): 8.24 (s, 1H), 8.03 (s, 1H), 7.42 (m, 6H), 7.23 (m, 4H), 7.09 (m, 3H), 7.01 (m, 4H), 6.90 (m, 5H), 5.27 (s, 2H), 4.47 (t, *J* = 8 Hz, 2H), 4.13 (t, *J* = 8 Hz, 2H), 1.99 (quint, *J* = 8 Hz, 2H), 1.70 (quint, *J* = 8 Hz, 2H).

Spectra Data S2 –Spectral Data – Sulfated Products

1a – Sodium 6-chloro-7-methyl-2-oxo-2*H*-chromen-4-yl sulfate – ^{1}H NMR (DMSO-d_6 400 MHz): 7.72 (s, 1H), 7.63 (s, 1H), 6.28 (s, 1H), 2.76 (s, 3H). ^{13}C NMR (DMSO-d_6, 100 MHz): 161.35, 159.75, 151.46, 128.79, 122.15, 118.89, 114.85, 96.21, 52.79, 19.86. MS (ESI) calculated for $C_{10}H_6ClNaO_6S$ $[(M\text{-}Na)]^-$, *m/z* 289.6519, found $[(M\text{-}Na)]^-$, *m/z* 289.4312.

1b – Sodium 3-chloro-4-methyl-2-oxo-2*H*-chromen-7-yl sulfate – ^{1}H NMR (DMSO-d_6 400 MHz): 7.81-7.79 (d, *J* = 8 Hz, 1H), 7.25-7.23 (d, *J* = 8 Hz, 2H), 2.57 (s, 3H). ^{13}C NMR (DMSO-d_6, 100 MHz): 156.74, 156.37, 151.67, 148.71, 126.38, 117.11, 116.78, 114.36, 106.58, 16.08. MS (ESI) calculated for $C_{10}H_6ClNaO_6S$ $[(M\text{-}Na)]^-$, *m/z* 289.6518, found $[(M\text{-}Na)]^-$, *m/z* 289.3933.

1c – Sodium 6-chloro-2-oxo-2*H*-chromen-7-yl sulfate – ^{1}H NMR (DMSO-d_6 400 MHz): 7.99-7.97 (d, *J* = 8 Hz, 1H), 7.87 (s, 1H), 7.59 (s, 1H), 6.43-6.41 (d, *J* = 8 Hz, 1H). ^{13}C NMR (DMSO-d_6, 100 MHz): 159.71, 152.74, 151.98, 143.13, 128.31, 119.64, 114.68, 114.41, 107.76. MS (ESI) calculated for $C_9H_4ClNaO_6S$ [(M-Na)]$^-$, *m/z* 275.6248, found [(M-Na)]$^-$, *m/z* 275.3681.

1d – Sodium 6-chloro-4-methyl-2-oxo-2*H*-chromen-7-yl sulfate– ^{1}H NMR (DMSO-d_6 400 MHz): 7.84 (s, 1H), 7.59 (s, 1H), 6.32 (s, 1H), 2.43 (s, 3H). ^{13}C NMR (DMSO-d_6, 100 MHz): 159.62,152.52, 152.11, 151.97, 125.60, 119.66, 115.31, 112.92, 107.73, 18.04. MS (ESI) calculated for $C_{10}H_6ClO_6S$ [(M-Na)]$^-$, *m/z* 289.6620, found [(M-Na)]$^-$, *m/z* 289.3933.

2a – Sodium 6-chloro-3-(3,4-dimethoxyphenyl)-4-methyl-2-oxo-2*H*-chromen-7-yl sulfate – ^{1}H NMR (DMSO-d_6, 400 MHz): 7.81 (s, 1H), 7.53 (s, 1H), 6.97-6.94 (d, *J* = 12 Hz 1H), 6.85 (s, 1H), 6.78-6.75 (d, J = 12 Hz, 1H), 3.73 (s, 3H), 3.67 (s, 3H), 2.20 (s, 3H). ^{13}C NMR (DMSO-d_6, 100M Hz): 159.80, 151.09, 148.32, 145.09, 126.76, 125.96, 124.84, 122.68, 119.67, 115.87, 114.05, 111.49, 107.46, 55.63, 55.53, 16.47. MS (ESI) calculated for $C_{18}H_{14}ClNaO_8S$ [(M-Na)]$^-$, *m/z* 425.8018, found [(M-Na)]$^-$, *m/z* 425.5155.

2b – Sodium 3-(2-chlorophenyl)-2-oxo-2*H*-chromen-7-yl sulfate – ^{1}H NMR (DMSO-d_6, 400 MHz): 8.00 (s, 1H), 7.63-7.61 (d, *J* = 8 Hz, 1H), 7.51-7.37 (m, 4H), 7.22 (s, 1H), 7.14-7.12 (d, *J* = 8 Hz, 1H). ^{13}C NMR (DMSO-d_6, 100M Hz): 159.00, 157.05, 154.21, 142.96, 134.26, 132.86, 131.82, 130.14, 129.26, 129.11, 127.19, 123.65, 116.82, 113.92. MS (ESI) calculated for $C_{15}H_8ClNaO_6S$ [(M-Na)]$^-$, *m/z* 351.7228, found [(M-Na)]$^-$, *m/z* 351.4232.

2c – Sodium 6-chloro-4-methyl-2-oxo-3-phenyl-2*H*-chromen-7-yl sulfate – ^{1}H NMR (DMSO-d_6, 400 MHz): 7.83 (s, 1H), 7.55 (s, 1H), 7.41-7.32 (m, 3H), 7.26-7.23 (d, *J* = 8 Hz, 2H), 2.17 (s, 3H). ^{13}C NMR (DMSO-d_6, 100M Hz): 159.71, 151.75, 151.18, 147.13,134.46, 130.19,130.12, 128.09, 127.87, 126.06, 124.92, 119.76, 115.77, 107.53, 16.39. MS (ESI) calculated for $C_{16}H_{10}ClNaO_6S$ [(M-Na)]$^-$, *m/z* 365.7498, found [(M-Na)]$^-$, *m/z* 365.4767.

2d – Sodium 3-(2-chlorophenyl)-2-oxo-4-phenyl-2*H*-chromen-7-yl sulfate – ^{1}H NMR (DMSO-d_6, 400 MHz): 7.38-7.11 (m, 12H), 7.00-6.98 (d, *J* = 8 Hz, 1H). ^{13}C NMR (DMSO-d_6, 100M Hz): 161.49, 159.42, 156.92, 154.68, 153.58, 133.80, 133.54, 132.65, 132.51, 129.58, 128.82, 128.64, 128.49, 128.30, 127.99, 127.20, 126.62, 116.87, 113.42, 106.96, 102.33. MS (ESI) calculated for $C_{21}H_{12}ClNaO_6S$ [(M-Na)]$^-$, *m/z* 427.8208, found [(M-Na)]$^-$, *m/z* 427.5336.

2e – Sodium 3-(3-chlorophenyl)-2-oxo-4-phenyl-2*H*-chromen-7-yl sulfate – ^{1}H NMR (DMSO-d_6, 400 MHz): 7.32-7.25 (m, 4H), 7.18-7.11 (m, 5H), 7.03-7.01 (dd, *J* = 8 Hz, 2H), 6.91-6.89 (d, *J* = 8 Hz, 1H). ^{13}C NMR (DMSO-d_6, 100M Hz): 177.67, 160.22, 156.83, 153.39, 151.66, 151.23, 136.60, 134.13, 132.00, 130.39, 129.37, 129.22, 128.96, 128.38, 128.23, 127.89, 127.19, 122.85, 116.62, 115.08, 106.80. MS (ESI) calculated for $C_{21}H_{12}ClNaO_6S$ [(M-Na)]$^-$, *m/z* 427.8208, found [(M-Na)]$^-$, *m/z* 427.5447.

2f – Sodium 3-(3-chlorophenyl)-4-methyl-2-oxo-2*H*-chromen-7-yl sulfate – ^{1}H NMR (DMSO-d_6, 400 MHz): 7.80-7.78 (d, *J* = 8 Hz, 1H), 7.52-7.43 (m, 3H), 7.31-7.29 (m, 1H), 7.26-7.21 (m, 2H), 2.26 (s, 3H). ^{13}C NMR (DMSO-d_6, 100M Hz): ^{13}C NMR (DMSO-d_6, 100M Hz): 159.89, 156.59, 152.83, 148.59, 139.05, 136.87, 132.71, 130.00, 129.06, 127.79, 126.50, 122.71,

116.40, 114.96, 106.48, 16.42. MS (ESI) calculated for $C_{16}H_{10}ClNaO_6S$ $[(M\text{-}Na)]^-$, *m/z* 365.7498, found $[(M\text{-}Na)]^-$, *m/z* 365.4838.

2g – Sodium 3-(4-chlorophenyl)-4-methyl-2-oxo-2*H*-chromen-6-yl sulfate – ^1H NMR (DMSO-*d6*, 400 MHz): 7.52-7.51 (d, *J* = 4 Hz, 1H), 7.47-7.45 (d, *J* = 8 Hz, 2H), 7.42-7.39 (dd, *J* = 8 Hz, 1H), 7.32-7.29 (d, *J* = 8 Hz, 3H), 2.16 (s, 3H). ^{13}C NMR (DMSO-*d6*, 100M Hz): 159.79, 149.83, 148.02, 147.97, 133.49, 132.69, 132.31, 132.09, 128.16, 125.19, 124.77, 120.06, 116.74, 116.63, 116.57, 16.44. MS (ESI) calculated for $C_{16}H_{10}ClNaO_6S$ $[(M\text{-}Na)]^-$, *m/z* 365.7498, found $[(M\text{-}Na)]^-$, *m/z* 365.4893.

2h – Sodium 3-(2-chlorophenyl)-4-methyl-2-oxo-2*H*-chromen-7-yl sulfate – ^1H NMR (DMSO-*d6*, 400 MHz): 7.74-7.72 (d, *J* = 8 Hz, 1H), 7.54-7.51 (m, 1H), 7.41-7.32 (m, 3H), 7.20-7.15 (m, 2H), 2.09 (s, 3H). ^{13}C NMR (DMSO-*d6*, 100M Hz): 159.10, 156.75, 153.02, 149.62, 133.63, 133.29, 132.04, 130.03, 129.23, 127.34, 126.51, 121.85, 116.55, 114.62, 106.64, 16.05. MS (ESI) calculated for $C_{16}H_{10}ClNaO_6S$ $[(M\text{-}Na)]^-$, *m/z* 365.7498, found $[(M\text{-}Na)]^-$, *m/z* 365.4880.

2i – Sodium 3-(2,4-dichlorophenyl)-4-methyl-2-oxo-2*H*-chromen-7-yl sulfate – ^1H NMR (DMSO-*d6*, 400 MHz): 7.72 (s, 1H), 7.54-7.33 (m, 5H), 2.09 (s, 3H). ^{13}C NMR (DMSO-*d6*, 100 MHz): 158.97, 156.96, 153.08, 150.10, 134.48, 133.76, 133.43, 132.75, 128.83, 127.60, 126.57, 120.77, 116.58, 114.49, 106.63, 16.07. MS (ESI) calculated for $C_{16}H_9Cl_2NaO_6S$ $[(M\text{-}Na)]^-$, *m/z* 400.1918, found $[(M\text{-}Na)]^-$, *m/z* 399.4618.

2j – Sodium 3-(2,4-dichlorophenyl)-4-methyl-2-oxo-2*H*-chromen-6-yl sulfate – ^1H NMR (DMSO-*d6*, 400 MHz): 7.72 (s, 1H), 7.54-7.33 (m, 5H), 2.08 (s, 3H). ^{13}C NMR (DMSO-*d6*, 100 MHz): 158.79, 154.01, 150.00, 149.72, 148.25, 134.28, 133.88, 133.24, 132.65, 128.85, 127.64, 125.32, 123.17, 119.53, 116.81, 16.10. MS (ESI) calculated for $C_{16}H_9Cl_2NaO_6S$ $[(M\text{-}Na)]^-$, *m/z* 400.1918, found $[(M\text{-}Na)]^-$, *m/z* 399.4697.

2k – Sodium 3-(2,4-dichlorophenyl)-2-oxo-4-phenyl-2*H*-chromen-7-yl sulfate – ^1H NMR (DMSO-*d6*, 400 MHz): 7.32–7.25 (m, 4H), 7.18–7.12 (m, 5H), 7.03–7.01 (m, 2H), 6.91-6.89 (d, *J* = 8 Hz, 1H). ^{13}C NMR (DMSO-*d6*, 100 MHz): 177.67, 170.10, 160.22, 156.83, 153.39, 151.66, 151.23, 136.60, 134.13, 132.00, 130.39, 129.37, 129.22, 128.96, 128.23, 127.89, 127.19, 122.85, 116.62, 115.08, 106.80. MS (ESI) calculated for $C_{21}H_{11}Cl_2NaO_6S$ $[(M\text{-}Na)]^-$, *m/z* 462.2628, found $[(M\text{-}Na)]^-$, *m/z* 461.0723.

2l – Sodium 3-(4-chlorophenyl)-2-oxo-4-phenyl-2*H*-chromen-7-yl sulfate – ^1H NMR (DMSO-*d6*, 400 MHz): 7.31-7.26 (m, 4H), 7.17-7.08 (m, 7H), 7.03-7.00 (d, *J* = 12 Hz, 1H), 6.91-6.88 (d, *J* = 12 Hz, 1H). ^{13}C NMR (DMSO-*d6*, 100 MHz): 161.23, 160.47, 159.94, 154.41, 151.94, 134.40, 133.53, 132.55, 132.52, 132.47, 131.78, 128.97, 128.76, 128.28, 128.22, 127.48, 127.43, 121.15, 113.27, 112.31, 102.20. MS (ESI) calculated for $C_{21}H_{12}ClNaO_6S$ $[(M\text{-}Na)]^-$, *m/z* 427.8208, found $[(M\text{-}Na)]^-$, *m/z* 427.5599.

2m – Sodium 3-(2,4-dichlorophenyl)-2-oxo-2*H*-chromen-7-yl sulfate – ^1H NMR (DMSO-*d6*, 400 MHz): 8.02 (s, 1H), 7.68 (s, 1H), 7.63-7.61 (d, *J* = 8 Hz, 1H), 7.48 (s, 1H), 7.47 (s, 1H), 7.22 (s, 1H), 7.15-7.12 (d, *J* = 8 Hz, 1H). ^{13}C NMR (DMSO-*d6*, 100 MHz): 158.88, 157.14, 154.25, 143.36, 133.96, 133.91, 133.25, 133.14, 129.26, 128.81, 127.42, 122.53, 116.86, 113.84, 106.55.

MS (ESI) calculated for $C_{15}H_7Cl_2NaO_6S$ $[(M-Na)]^-$, *m/z* 386.1648, found $[(M-Na)]^-$, *m/z* 385.4435.

2n – Sodium 3-(4-bromophenyl)-2-oxo-2*H*-chromen-7-yl sulfate – ^{1}H NMR (DMSO-*d*$_6$, 400 MHz): 8.20 (s, 1H), 7.65-7.57 (m, 5H), 7.19 (s, 1H), 7.13-7.10 (d, *J* = 8 Hz, 1H). ^{13}C NMR (DMSO-*d*$_6$, 100 MHz): 159.66, 156.87, 153.86, 140.92, 134.08, 131.12, 130.43, 129.15, 122.95, 121.57, 116.76, 114.50, 106.28, 104.80, 99.49. MS (ESI) calculated for $C_{15}H_8BrNaO_6S$ $[(M-Na)]^-$, *m/z* 396.1768, found $[(M-Na)]^-$, *m/z* 397.3833.

2o – Sodium 3-(4-bromophenyl)-2-oxo-4-phenyl-2*H*-chromen-7-yl sulfate – ^{1}H NMR (DMSO-*d*$_6$ 400 MHz): 7.40-7.36 (m, 6H), 7.23-7.21 (d, *J* = 8 Hz, 2H), 7.12-7.08 (m, 3H), 6.98-6.96 (d, *J* = 8 Hz, 1H). ^{13}C NMR (DMSO-*d*$_6$, 100 MHz): 179.59, 160.26, 156.73, 153.33, 151.40, 144.49, 134.22, 133.79, 133.00, 132.79, 130.40, 129.00, 128.37, 128.27, 127.84, 123.08, 120.61, 116.60, 115.13, 106.79, 90.08. MS (ESI) calculated for $C_{21}H_{12}BrNaO_6S$ $[(M-Na)]^-$, *m/z* 472.2748, found $[(M-Na)]^-$, *m/z* 471.5574.

2p – Sodium 4-(4-chlorobenzyl)-2-oxo-3-phenyl-2*H*-chromen-7-yl sulfate – ^{1}H NMR (DMSO-*d*$_6$ 400 MHz): 7.42-7.39 (d, *J* = 12 Hz, 1H), 7.36-7.28 (m, 3H), 7.26-7.18 (m, 5H), 7.11-7.09 (d, *J* = 8 Hz, 2H), 6.99-6.96 (dd, *J* = 10, 4 Hz, 1H), 3.97 (s, 2H). ^{13}C NMR (DMSO-*d*$_6$, 100 MHz): 173.47, 160.75, 160.43, 154.47, 148.44, 137.10, 134.73, 134.62, 130.94, 129.78, 129.71, 128.49, 128.20, 127.85, 127.80, 124.36, 113.05, 111.10, 102.16, 99.38, 44.22, 33.95. MS (ESI) calculated for $C_{22}H_{14}ClNaO_6S$ $[(M-Na)]^-$, *m/z* 441.8478, found $[(M-Na)]^-$, *m/z* 441.6260.

2q – Sodium 3-(4-bromophenyl)-4-methyl-2-oxo-2*H*-chromen-7-yl sulfate – ^{1}H NMR (DMSO-*d*$_6$ 400 MHz): 7.80-7.78 (d, *J* = 8 Hz, 1H), 7.67-7.65 (d, *J* = 8 Hz, 2H), 7.32-7.30 (d, *J* = 8 Hz, 2H), 7.25-7.22 (m, 2H), 2.26 (s, 3H). ^{13}C NMR (DMSO-*d*$_6$, 100 MHz): 159.89, 156.58, 155.99, 152.81, 148.33, 134.00, 132.52, 131.06, 126.44, 122.89, 121.16, 116.39, 115.01, 106.48, 101.41, 16.40. MS (ESI) calculated for $C_{16}H_{10}BrNaO_6S$ $[(M-Na)]^-$, *m/z* 410.2038, found $[(M-Na)]^-$, *m/z* 409.4458.

2r – Sodium 3-(3-bromophenyl)-2-oxo-2*H*-chromen-7-yl sulfate – ^{1}H NMR (DMSO-*d*$_6$ 400 MHz): 8.31 (s, 1H), 7.96 (s, 1H), 7.77-7.75 (d, *J* = 8 Hz, 1H), 7.72-7.70 (d, *J* = 8 Hz, 1H), 7.62-7.60 (d, *J* = 8 Hz, 1H), 7.46-7.42 (t, *J* = 8 Hz, 1H), 7.27 (s, 1H), 7.21-7.19 (d, *J* = 8 Hz, 1H). ^{13}C NMR (DMSO-*d*$_6$, 100 MHz): 159.65, 157.05, 153.95, 141.49, 137.70, 137.21, 130.93, 130.88, 130.30, 129.25, 127.40, 122.53, 121.41, 116.79, 114.43, 106.28. MS (ESI) calculated for $C_{15}H_8BrNaO_6S$ $[(M-Na)]^-$, *m/z* 396.1768, found $[(M-Na)]^-$, *m/z* 397.4242.

2s – Sodium 3-(3-chlorophenyl)-4-methyl-2-oxo-2*H*-chromen-6-yl sulfate – ^{1}H NMR (DMSO-*d*$_6$ 400 MHz): 7.61-7.59 (m, 1H), 7.50-7.48 (m, 3H), 7.45 (s, 1H), 7.40-7.37 (m, 1H), 7.33-7.31 (m, 1H). 2.24 (s, 3H). ^{13}C NMR (DMSO-*d*$_6$, 100 MHz): 159.71, 149.90, 148.17, 148.05, 136.80, 132.75, 130.02, 129.87, 128.93, 127.92, 125.04, 124.84, 120.01, 116.73, 116.64, 44.20, 16.44. MS (ESI) calculated for $C_{16}H_{10}ClNaO_6S$ $[(M-Na)]^-$, *m/z* 365.7498, found $[(M-Na)]^-$, *m/z* 365.4892.

2t – Sodium 3-(4-chlorophenyl)-2-oxo-2*H*-chromen-7-yl sulfate – ^{1}H NMR (DMSO-*d*$_6$ 400 MHz): 8.27 (s, 1H), 7.79-7.77 (d, *J* = 8 Hz, 2H), 7.71-7.69 (d, *J* = 8 Hz, 1H), 7.54-7.52 (d, *J* = 8 Hz, 2H), 7.27 (s, 1H), 7.21-7.19 (d, *J* = 8 Hz, 1H). ^{13}C NMR (DMSO-*d*$_6$, 100 MHz): 167.40,

159.72, 156.85, 153.86, 140.95, 133.70, 132.94, 130.14, 129.15, 128.19, 122.90, 116.76, 114.50, 106.28, 44.21. MS (ESI) calculated for $C_{15}H_8ClNaO_6S$ [(M-Na)]$^-$, *m/z* 351.7228, found [(M-Na)]$^-$, *m/z* 351.4640.

2u – Sodium 3-(3-chlorophenyl)-2-oxo-2*H*-chromen-7-yl sulfate – ^{1}H NMR (DMSO-*d*$_6$ 400 MHz): 8.33 (s, 1H), 7.83 (s, 1H), 7.73-7.69 (m, 2H), 7.53-7.49 (m, 2H), 7.28 (s, 1H), 7.22-7.20 (d, *J* = 8 Hz, 1H). ^{13}C NMR (DMSO-*d*$_6$, 100 MHz): 159.65, 157.00, 153.93, 141.50, 136.94, 132.87, 131.07, 130.04, 129.26, 128.06, 127.01, 122.59, 116.77, 114.44, 106.26. MS (ESI) calculated for $C_{15}H_8ClNaO_6S$ [(M-Na)]$^-$, *m/z* 351.7228, found [(M-Na)]$^-$, *m/z* 351.4640.

2v – Sodium 4-(6-bromo-4-methyl-2-oxo-2*H*-chromen-3-yl)phenyl sulfate – ^{1}H NMR (DMSO-*d*$_6$ 400 MHz): 7.94 (s, 1H), 7.73 (dd, *J* = 8 Hz, 1H), 7.36 (d, *J* = 8 Hz, 1H), 7.17 (m, 4H), 2.22 (s, 3H). ^{13}C NMR (DMSO-*d*$_6$, 100 MHz): 159.46, 153.39, 151.06, 146.67, 133.93, 130.64, 128.48, 128.14, 126.97, 122.18, 119.88, 119.83, 118.45, 116.31, 26.17, 16.49. MS (ESI) calculated for $C_{16}H_{10}BrNaO_6S$ [(M-Na)]$^-$, *m/z* 410.2038, found [(M-Na)]$^-$, *m/z* 409.4867.

2w – Sodium 2-hydroxy-4-(2-oxo-4-phenyl-7-(sulfonatooxy)-2*H*-chromen-3-yl)phenyl sulfate – ^{1}H NMR (DMSO-*d*$_6$ 400 MHz): 7.38-7.31 (m, 5H), 7.20 (d, *J* = 8 Hz, 2H), 7.07 (dd, *J* = 8 Hz, 2.32 Hz, 1H), 6.93 (d, *J* = 8 Hz, 1H), 6.64 (dd, *J* = 8 Hz, 2.16 Hz, 1H). ^{13}C NMR (DMSO-*d*$_6$, 100 MHz): 160.46, 156.33, 153.16, 150.81, 143.27, 142.42, 134.41, 129.02, 128.97, 128.20, 128.13, 127.76, 127.47, 124.48, 123.98, 122.72, 118.27, 116.40, 115.30, 106.67, 99.49. MS (ESI) calculated for $C_{21}H_{12}Na_2O_{11}S_2$ [(M-2Na)]$^-$, *m/z* 504.4155, found [(M-2Na)]$^{2-}$, *m/z* 252.5107.

2x – Sodium 4-(6,8-dichloro-2-oxo-4-phenyl-2*H*-chromen-3-yl)phenyl sulfate – ^{1}H NMR (DMSO-*d*$_6$ 400 MHz): 8.02 (d, *J* = 2.4 Hz, 1H), 7.44-7.37 (m, 3H), 7.25-7.23 (m, 2H), 7.07 (d, *J* = 8 Hz, 2H), 7.00 (d, *J* = 8 Hz, 2H), 6.86 (d, *J* = 8 Hz, 1H). ^{13}C NMR (DMSO-*d*$_6$, 100 MHz): 160.44, 154.36, 150.33, 148.27, 134.89, 132.31, 132.21, 131.93, 130.36, 130.01, 129.88, 129.83, 129.45, 129.24, 128.95, 126.46, 124.46, 123.26, 122.66, 120.26, 120.20. MS (ESI) calculated for $C_{21}H_{11}Cl_2NaO_6S$ [(M-Na)]$^-$, *m/z* 462.2628, found [(M-Na)]$^-$, *m/z* 461.5803.

2y – Sodium 4-(2-oxo-4-phenyl-7-(sulfonatooxy)-2*H*-chromen-3-yl)phenyl sulfate – ^{1}H NMR (DMSO-*d*$_6$ 400 MHz): 7.40-7.31 (m, 4H), 7.23-7.21 (m, 2H), 7.08-6.93 (m, 6H). ^{13}C NMR (DMSO-*d*$_6$, 100 MHz): 160.62, 156.40, 153.15, 152.58, 150.83, 147.52, 134.60, 131.12, 129.04, 128.55, 128.23, 128.18, 127.71, 123.88, 118.91, 118.89, 118.82, 116.49, 115.36, 114.40, 106.74. MS (ESI) calculated for $C_{21}H_{12}Na_2O_{10}S_2$ [(M-2Na)]$^-$, *m/z* 488.4165, found [(M-2Na)]$^{2-}$, *m/z* 244.5372.

3a – Sodium 4-(2-oxo-7-((1-(3-((2-oxo-4-phenyl-3-(4-(sulfonatooxy)phenyl)-2*H*-chromen-7-yl)oxy)propyl)-1*H*-1,2,3-triazol-4-yl)methoxy)-4-phenyl-2*H*-chromen-3-yl)phenyl sulfate – ^{1}H NMR (DMSO-*d*$_6$ 400 MHz): 8.37 (s, 1H), 7.40-7.32 (m, 6H), 7.29 (s, 1H), 7.22-7.19 (m, 4H), 7.11 (s, 1H), 7.04-7.01 (m, 4H), 6.97-6.93 (m, 7H), 6.88 (dd, *J* = 8 Hz, 2.4 Hz, 1H), 5.29 (s, 2H), 4.61 (t, 2H), 4.16 (t, 2H), 2.35 (quint, *J* = 8 Hz, 2H). ^{13}C NMR (DMSO-*d*$_6$, 100 MHz): 193.62, 161.07, 160.73, 160.64, 154.10, 154.06, 152.58, 150.96, 150.94, 147.96, 143.19, 141.93, 134.61, 131.14, 129.02, 128.50, 128.38, 128.36, 128.24, 128.19, 127.46, 125.00, 123.60, 123.13, 123.02, 122.63, 118.84, 114.19, 113.92, 113.81, 112.85, 112.65, 102.77, 101.47, 101.18, 99.50, 67.38,

65.51, 65.49, 59.27, 52.77, 50.97, 48.67, 46.63, 46.59, 42.40, 29.22, 9.92. MS (ESI) calculated for $C_{48}H_{33}N_3Na_2O_{14}S_2$ $[(M-2Na)]^-$, *m/z* 939.8985, found $[(M-2Na)]^{2-}$, *m/z* 470.4533.

3b – Sodium 4-(2-oxo-7-((1-(4-((2-oxo-4-phenyl-3-(4-(sulfonatooxy)phenyl)-2*H*-chromen-7-yl)oxy)butyl)-1*H*-1,2,3-triazol-4-yl)methoxy)-4-phenyl-2*H*-chromen-3-yl)phenyl sulfate – ^{1}H NMR (DMSO-d_6 400 MHz): 8.35 (s, 1H), 7.40-7.31 (m, 6H), 7.29 (s, 1H), 7.21-7.19 (m, 4H), 7.11 (s, 1H), 7.04-7.01 (m, 4H), 6.97-6.94 (m, 4H), 6.92 (s, 1H), 6.90 (dd, *J* = 8 Hz, 2.36 Hz, 1H), 5.29 (s, 2H), 4.50 (t, *J* = 8 Hz, 2H), 4.15 (t, *J* = 8 Hz, 2H), 2.05 (quint, *J* = 8 Hz, 2H), 1.78 (quint, *J* = 8 Hz, 2H). ^{13}C NMR (DMSO-d_6, 100 MHz): 203.94, 196.89, 195.80, 175.54, 166.72, 165.45, 161.34, 161.31, 160.73, 157.49, 155.67, 154.06, 152.59, 141.90, 140.87, 139.56, 134.65, 134.61, 131.14, 129.52, 129.22, 129.15, 129.12, 129.03, 128.61, 128.52, 128.50, 128.45, 128.36, 128.31, 128.24, 124.82, 123.13, 120.66, 118.93, 118.83, 113.93, 112.68, 101.52, 101.12, 94.16, 88.00, 67.65, 61.78, 52.77, 51.47, 49.08, 26.41, 7.31. MS (ESI) calculated for $C_{49}H_{35}N_3Na_2O_{14}S_2$ $[(M-2Na)]^-$, *m/z* 953.9255, found $[(M-2Na)]^{2-}$, *m/z* 477.4864.

3c – Sodium 4-(2-oxo-7-((1-(3-((2-oxo-3-(4-(sulfonatooxy)phenyl)-2*H*-chromen-7-yl)oxy)propyl)-1*H*-1,2,3-triazol-4-yl)methoxy)-2*H*-chromen-3-yl)phenyl sulfate – ^{1}H NMR (DMSO-d_6 400 MHz): 8.38 (s, 1H), 8.18 (s, 2H), 7.72-7.62 (m, 6H), 7.25-7.22 (m, 5H), 7.05-7.04 (m, 2H), 6.99 (dd, *J* = 8 Hz, 2.4 Hz, 1H), 5.30 (s, 2H), 4.62 (t, *J* = 8 Hz, 2H), 4.17 (t, *J* = 8 Hz, 2H), 2.37 (quint, *J* = 8 Hz, 2H). ^{13}C NMR (DMSO-d_6, 100 MHz): 175.55, 159.13, 144.57, 139.90, 139.49, 136.17, 135.28, 132.43, 131.38, 128.92, 128.85, 126.18, 123.16, 122.63, 119.91, 117.12, 116.36, 115.79, 110.72, 109.93, 108.24, 103.12, 102.74, 88.62, 79.35, 78.57, 72.02, 71.32, 66.46, 56.09, 47.12, 42.14, 40.72, 37.56, 13.22, 10.00. MS (ESI) calculated for $C_{36}H_{25}N_3Na_2O_{14}S_2$ $[(M-2Na)]^-$, *m/z* 787.7025, found $[(M-2Na)]^{2-}$, *m/z* 394.2348.

3d – Sodium 4-(2-oxo-7-((1-(4-((2-oxo-3-(4-(sulfonatooxy)phenyl)-2*H*-chromen-7-yl)oxy)butyl)-1*H*-1,2,3-triazol-4-yl)methoxy)-2*H*-chromen-3-yl)phenyl sulfate – ^{1}H NMR (DMSO-d_6 400 MHz): 8.35 (s, 1H), 8.18 (s, 2H), 7.71-7.62 (m, 6H), 7.25-7.22 (m, 5H), 7.04-6.97 (m, 3H), 5.29 (s, 2H), 4.51 (t, *J* = 8 Hz, 2H), 4.16 (t, *J* = 8 Hz, 2H), 2.06 (quint, *J* = 8 Hz, 2H), 1.79 (quint, *J* = 8 Hz, 2H). ^{13}C NMR (DMSO-d_6, 100 MHz): 181.55, 175.12, 174.05, 168.82, 160.00, 154.61, 154.52, 145.95, 141.93, 141.60, 139.90, 129.56, 129.39, 128.85, 124.83, 123.15, 122.93, 119.91, 113.39, 113.10, 112.90, 105.82, 101.07, 100.68, 94.85, 67.61, 61.76, 57.57, 49.07, 45.69, 44.08, 26.43, 25.47, 18.59, 13.88, 7.98, 3.25. MS (ESI) calculated for $C_{37}H_{27}N_3Na_2O_{14}S_2$ $[(M-2Na)]^-$, *m/z* 801.7295, found $[(M-2Na)]^{2-}$, *m/z* 401.2678.

3e – Sodium 4-(4-methyl-7-((1-(3-((4-methyl-2-oxo-3-(4-(sulfonatooxy)phenyl)-2*H*-chromen-7-yl)oxy)propyl)-1*H*-1,2,3-triazol-4-yl)methoxy)-2-oxo-2*H*-chromen-3-yl)phenyl sulfate – ^{1}H NMR (DMSO-d_6 400 MHz): 8.38 (s, 1H), 7.79 (d, *J* = 8 Hz, 1H) 7.76 (d, *J* = 8 Hz, 1H), 7.26-7.20 (m, 8H), 7.09-6.97 (m, 4H), 5.31 (s, 2H), 4.63 (t, *J* = 8 Hz, 2H), 4.17 (t, *J* = 8 Hz, 2H), 2.27 (s, 6H), 2.40 (quint, *J* = 8 Hz, 2H). ^{13}C NMR (DMSO-d_6, 100 MHz): 161.19, 160.63, 160.22, 153.66, 153.58, 153.06, 147.91, 147.87, 141.98, 130.79, 129.05, 127.00, 126.96, 124.80, 123.09, 122.90, 119.84, 113.84, 113.57, 112.68, 112.48, 101.33, 100.93, 84.69, 67.56, 61.73, 56.57, 52.78, 49.09, 44.34, 36.61, 26.59, 26.44, 25.48, 16.44, 13.45, 10.45, 3.42. MS (ESI) calculated for $C_{38}H_{29}N_3Na_2O_{14}S_2$ $[(M-2Na)]^-$, *m/z* 815.7565, found $[(M-2Na)]^{2-}$, *m/z* 408.7097.

3f – Sodium 4-(4-methyl-7-((1-(4-((4-methyl-2-oxo-3-(4-(sulfonatooxy)phenyl)-2*H*-chromen-7-yl)oxy)butyl)-1*H*-1,2,3-triazol-4-yl)methoxy)-2-oxo-2*H*-chromen-3-yl)phenyl sulfate – ^{1}H

NMR (DMSO-d_6 400 MHz): 8.36 (s, 1H), 7.79 (m, 2H), 7.22-7.19 (m, 9H), 7.09-6.98 (m, 3H), 5.30 (s, 2H), 4.52 (t, J = 8 Hz, 2H), 4.16 (t, J = 8 Hz, 2H), 2.27 (s, 6H), 2.07 (quint, J = 8 Hz, 2H), 1.79 (quint, J = 8 Hz, 2H). ^{13}C NMR (DMSO-d_6, 100 MHz): 168.27, 166.21, 161.19, 160.63, 160.22, 153.66, 153.58, 153.06, 147.91, 147.87, 141.98, 130.79, 129.05, 127.00, 126.96, 124.80, 123.09, 122.90, 119.84, 113.84, 113.57, 112.68, 112.48, 101.33, 100.93, 84.69, 82.62, 67.56, 61.73, 56.57, 52.78, 49.09, 44.34, 26.59, 26.44, 25.48, 16.44, 13.45, 3.42. MS (ESI) calculated for $C_{39}H_{31}N_3Na_2O_{14}S_2$ [(M-2Na)]$^-$, *m/z* 829.7835, found [(M-2Na)]$^{2-}$, *m/z* 415.2930.

3g – Sodium 4-(7-((1-(4-((3-(2,4-dichlorophenyl)-2-oxo-4-phenyl-2*H*-chromen-7-yl)oxy)butyl)-1*H*-1,2,3-triazol-4-yl)methoxy)-2-oxo-4-phenyl-2*H*-chromen-3-yl)phenyl sulfate – ^{1}H NMR (DMSO-d_6 400 MHz): 8.24 (s, 1H), 8.03 (s, 1H), 7.41-7.34 (m, 6H), 7.24-7.18 (m, 4H), 7.10-7.08 (m, 3H), 7.03-6.94 (m, 4H), 6.91-6.86 (m, 5H), 5.07 (s, 2H), 4.46 (t, J = 8 Hz, 2H), 4.11 (t, J = 8 Hz, 2H), 1.99 (quint, J = 8 Hz, 2H), 1.71 (quint, J = 8 Hz, 2H). ^{13}C NMR (DMSO-d_6, 100 MHz): 185.99, 161.31, 160.64, 159.15, 154.45, 154.14, 150.97, 148.97, 146.95, 142.47, 140.96, 134.63, 133.65, 131.67, 131.45, 131.12, 130.60, 130.11, 129.14, 129.07, 129.02, 128.63, 128.49, 128.33, 128.23, 127.96, 125.11, 124.46, 123,12, 122.95, 121.36, 119.36, 118.82, 114.16, 113.77, 112.64, 104.29, 101.20, 68.03, 67.65, 61.02, 56.52, 49.03, 47.23, 45.67, 26.37, 25.45, 16.06, 5.86. MS (ESI) calculated for $C_{49}H_{34}Cl_2N_3NaO_{10}S$ [(M-Na)]$^-$, *m/z* 927.7718, found [(M-Na)]$^-$, *m/z* 928.8280.

Figure S7 – Antithrombin – FXa Inactivation Study

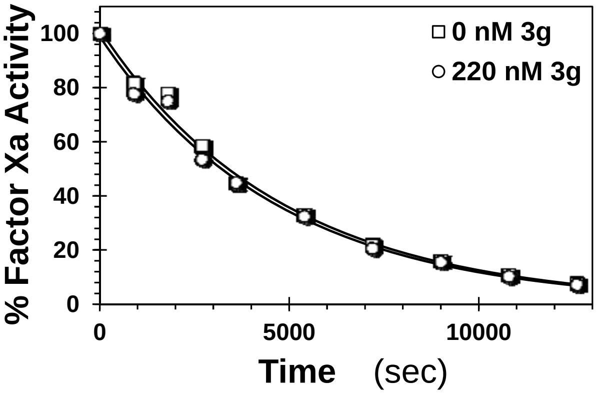

Figure S7: Residual factor Xa activity as a function of time following incubation with excess antithrombin in the presence of a fixed concentration of **3g** (0 or 220 nM) in pH 7.2 buffer at 37 °C. Solid lines represent exponential fits to the data using equation 6 to calculate the pseudo first order rate constant k_{OBS}.
